# Relativistic Hirshfeld atom refinement of an organo-gold(I) compound

**DOI:** 10.1107/S2052252521004541

**Published:** 2021-05-26

**Authors:** Sylwia Pawlędzio, Maura Malinska, Magdalena Woińska, Jakub Wojciechowski, Lorraine Andrade Malaspina, Florian Kleemiss, Simon Grabowsky, Krzysztof Woźniak

**Affiliations:** a Biological and Chemical Research Centre, Department of Chemistry, University of Warsaw, Żwirki i Wigury 101, Warszawa 02-089, Poland; b Rigaku Europe SE, Hugenottenallee 167, 63263 Neu-Isenburg, Germany; c Department of Chemistry, Biochemistry and Pharmaceutical Sciences, University of Bern, Freiestrasse 3, Bern 3012, Switzerland

**Keywords:** Hirshfeld atom refinement, relativistic effects, aspherical atom model

## Abstract

The main goal of this study is the validation of relativistic Hirshfeld atom refinement (HAR) as implemented in *Tonto* for high-resolution X-ray diffraction datasets of an organo-gold(I) compound. The influence of the relativistic effects on statistical parameters, geometries and electron density properties was analyzed and compared with the influence of electron correlation and anharmonic atomic motions. Recent work in this field has indicated the importance of relativistic effects in the static electron density distribution of organo-mercury compounds. This study confirms that differences in electron density due to relativistic effects are also of significant magnitude for organo-gold compounds. Relativistic effects dominate not only the core region of the gold atom, but also influence the electron density in the valence and bonding region, which has measurable consequences for the HAR refinement model parameters. To study the effects of anharmonic motion on the electron density distribution, dynamic electron density difference maps were constructed. Unlike relativistic and electron correlation effects, the effects of anharmonic nuclear motion are mostly observed in the core area of the gold atom.

## Abstract 2

A series of Hirshfeld atom refinements (HARs) has been performed for an organo-gold(I) compound at different levels of theory. The influences of the relativistic effect, electron correlation and anharmonic thermal motion have been studied based on aspherical charge density models obtained with HAR.

## 1. Introduction   

After the famous Dirac statement (Dirac & Fowler, 1929 ▸) saying that relativistic effects are of ‘no importance in the consideration of atomic and molecular structure and ordinary chemical reactions’, it took nearly half a century to find and confirm important influences of relativistic effects on the electronic structure of compounds (Grant, 1970 ▸; Desclaux, 1973 ▸; Ziegler *et al.*, 1981 ▸). During the last 50 years, relativistic quantum chemistry has undergone significant development and methodological progress; nowadays, it is well known that a relativistic quantum formalism is necessary in the study of compounds with heavy elements (Desclaux & Pyykkö, 1976 ▸; Pyykkö, 1988 ▸; van Lenthe *et al.*, 1996 ▸; Reiher & Wolf, 2004 ▸; Baková *et al.*, 2011 ▸). Relativistic effects appear when the speed of electrons approaches the speed of light. For valence shells, the effect increases with *Z* ^2^, where *Z* is the atomic number of the heavy element (Pyykkö, 1988 ▸). Quantitively it is rather small, but it can cause changes in the chemical behavior of elements within the same group (Desclaux & Pyykkö, 1976 ▸). For heavy elements (with *Z* > 50) (Onoe, 2000 ▸) the magnitude of the relativistic effects becomes high enough to strongly influence the chemical and physical properties of crystals, which has been reported several times (Schwerdtfeger, 2002 ▸; Christensen & Seraphin, 1971 ▸; Pitzer, 1979 ▸). Well known examples include the yellow color of gold (Pyykkö, 1988 ▸), the low melting temperature of mercury (Pyykkö, 1988 ▸) and the high voltage of lead-acid car batteries (about 80% of its voltage comes from relativistic effects) (Ahuja *et al.*, 2011 ▸). Relativistic effects expand the chemistry of gold beyond the standard chemistry of a ‘coinage metal’ to that of a ‘noble metal’. Owing to the high stability of the 6*s* orbitals, gold is able to form aurides with the alkali metals (*e.g.* Cs, Rb), where it has an atypical oxidation state for group 11 elements of −1 (Jansen, 2005 ▸). Moreover, closed-shell aurophilic interactions were found in gold nanoclusters with energies comparable to those of hydrogen bonds (Pyykkö, 1997 ▸; Bardají & Laguna, 1999 ▸; Codina *et al.*, 2002 ▸).

Since the electron density is an observable, it can be used to investigate how relativistic effects manifest themselves in the electronic structure of heavy-metal compounds and is of particular interest for both experimental and theoretical studies (Zuo *et al.*, 1999 ▸). X-ray diffraction (XRD) experiments yield structure-factor modules that include information about the electron density which can be extracted in well known refinement procedures (Coppens, 1997 ▸). Thus, high-resolution single-crystal XRD has become a very convenient experimental technique for topological analysis of electron density distributions of molecules in crystals (Bader, 1994 ▸; Gatti, 2005 ▸; Tsirelson & Ozerov, 1996 ▸; Farrugia *et al.*, 2009 ▸; Koritsanszky & Coppens, 2001 ▸). However, the treatment of heavy elements from an experimental point of view is not a trivial task, because of difficulties arising from high X-ray absorption (Als-Nielsen & McMorrow, 2011 ▸), extinction (Chandrasekhar, 1960 ▸), anomalous dispersion (Caticha-Ellis, 1981 ▸), partial disorder (Destro *et al.*, 2017 ▸), anharmonic thermal motions (Herbst-Irmer *et al.*, 2013 ▸) or sample decay (Christensen *et al.*, 2019 ▸). It requires sophisticated data collection and reduction procedures, as well as advanced methods describing the electron density distribution. On the other hand, all-electron relativistic quantum mechanical calculations (Pantazis & Neese, 2014 ▸; Smith, 2003 ▸; Reiher, 2012 ▸) are time-consuming, require dedicated software and need to take into account other effects, such as electron correlation (Matito *et al.*, 2013 ▸). Despite the above-mentioned difficulties, recent studies suggest that relativistic effects can be detected from high-resolution and high-quality XRD experiments (Bučinský *et al.*, 2016 ▸; Hudák *et al.*, 2010 ▸; Eickerling *et al.*, 2007 ▸; Batke & Eickerling, 2016 ▸) and modeled by applying quantum crystallography methods.

In quantum crystallography, the most widely applicable model describing the aspherical distribution of electron density is the Hansen–Coppens multipole model (Hansen & Coppens, 1978 ▸). In the standard multipolar model, electron density is modeled as the sum of pseudoatoms. The density of a pseudoatom is generated from the sum of the spherical electron density of the frozen-core and the normalized valence density, which is described by normalized Slater functions. Expansion–contraction of the spherical and multipolar valence density is described by the parameters κ and κ′, respectively. During least-squares refinement, the static population and expansion–contraction parameters are optimized together with atomic positions and with their anisotropic displacement parameters (ADPs) against experimental structure factors. In this approach, each multipole is modeled separately (Coppens, 1997 ▸). In the method of the extended Hansen–Coppens multipole model, each atomic shell can be treated separately, which should provide sufficient refinement flexibility (Zhurov *et al.*, 2011 ▸; Fischer *et al.*, 2011 ▸). Unfortunately, even then heavy elements are problematic for the multipolar model (Stalke, 2012 ▸). In addition, the multipole model in general faces a lot of difficulties, *e.g.* high correlation between refined parameters, high residual density, issues with overparameterization, overfitting and the requirement for very high quality diffraction data (Gianopoulos *et al.*, 2017 ▸). Due to the above-mentioned restrictions, limited works concerning electron density studies of organometallic compounds are present in the literature (Maslen *et al.*, 1994 ▸, 1995 ▸; du Boulay *et al.*, 1995 ▸; Iversen *et al.*, 1998 ▸, 1999 ▸; Schiøtt *et al.*, 2004 ▸; Coppens *et al.*, 2005 ▸; Poulsen *et al.*, 2007 ▸; Kamiński *et al.*, 2011 ▸; Gianopoulos *et al.*, 2017 ▸; Zhurov *et al.*, 2011 ▸; Pawlędzio *et al.*, 2020 ▸); however, they do not describe relativistic effects explicitly.

**Figure 1:**
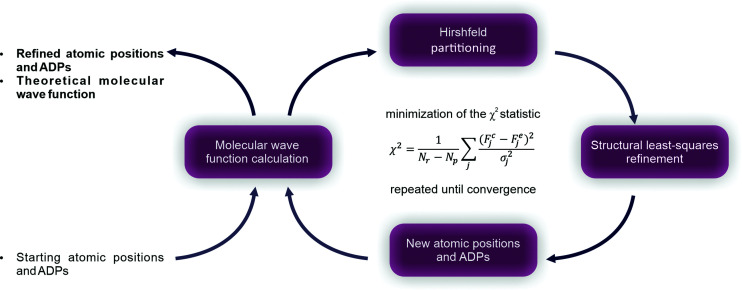
Scheme of Hirshfeld atom refinement.

In 2008, Jayatilaka and Dittrich introduced Hirshfeld atom refinement (HAR), which allows non-spherical atomic form factor calculations using quantum-mechanical methods (Jayatilaka & Dittrich, 2008 ▸). Later, Capelli *et al.* (2014 ▸) extended the original HAR and implemented an iterative refinement procedure. The first step in HAR is an *ab initio* quantum mechanical calculation of the molecular electron density using Hartee–Fock (HF) or density functional theory (DFT). The theoretical molecular electron density is then divided (Stockholder partitioning) (Hirshfeld, 1977*a* ▸,*b* ▸) into aspherical atomic electron densities (Hirshfeld atoms). As a result, tailor-made Hirshfeld atomic scattering factors are calculated and used to refine structural parameters (atomic coordinates and ADPs) against the measured structure factors (Fig. 1 ▸). HAR offers full flexibility in the calculation of the molecular electron density in the context of choosing the method and basis set. This procedure is becoming an increasingly popular technique for structure refinement (Fugel *et al.*, 2014 ▸; Woińska *et al.*, 2016 ▸, 2017 ▸; Malaspina *et al.*, 2019 ▸; Chodkiewicz *et al.*, 2020 ▸; Kleemiss *et al.*, 2021 ▸).

The above mentioned quantum crystallography method (HAR) has been implemented in the *Tonto* (Jayatilaka & Grimwood, 2001 ▸) package, and the relativistic effects at the infinite-order two-component (IOTC) level of theory were introduced to *Tonto* in 2010 (Bučinský *et al.*, 2010 ▸). The subsequent series of papers that followed studied the impact of relativity on electron density, Laplacian and Fourier transform of heavy atoms and transition metal complexes (Bučinský *et al.*, 2012 ▸, 2011 ▸, 2014 ▸). In 2016, Bučinský *et al.* (2016 ▸) used the IOTC approach in *Tonto* to present the first relativistic HAR and demonstrate the impact of relativistic effects and electron correlation on electron density and structure factors of di­phenyl mercury (HgPh_2_) and tri­phenyl bis­muth (BiPh_3_) at the BLYP level of theory. They found that relativistic effects are important not only in the core electron density of metal atoms, but are also significant in the outer core and bonding regions. In 2019, Bučinský *et al.* (2019 ▸) validated relativistic HAR against theoretical structure factors and discussed many physical properties (*e.g.* electron correlation, thermal motion or crystalline environment) which could be accounted for by the more accurate HAR. They summarized the size of tested effects as follows: relativity >> electron correlation > ADP model > basis set ∼ crystalline environment.

There have been many theoretical studies that discuss the importance of relativistic effects and electron correlation on the electron densities. For example, Eickerling *et al.* (2007 ▸) performed a systematic study on the topology of the electron density distribution for different relativistic approaches on compounds containing Ni, Pd and Pt. They showed that differences in topological parameters are crucial at bond critical points (BCPs), which proves that relativistic effects are significant in the bonding region. Several studies concentrate on the quantum theory of atoms in molecules (QTAIM) analysis of electron density obtained at different levels of relativistic and quasi-relativistic theories, contrasting relativistic and non-relativistic approaches (Christensen & Seraphin, 1971 ▸; Echeverría *et al.*, 2015 ▸; Anderson *et al.*, 2019 ▸).

The aim of this study is to show the influence of the IOTC implementation of HAR performed against experimental structure factors for three different datasets measured with Ag, Mo and synchrotron radiation (λ = 0.2486 Å) of an organo-gold(I) crystal structure in terms of data quality and crystallographic statistical indicators. The final charge density models are used to examine changes in the electron density arising from relativistic effects, electron correlation and anharmonic motions of the gold atom. The comparison to anharmonic motion required some method development to be able to output and subtract dynamic electron density grid files. Hence, the examination of the magnitude of the effect of relativistic and electron correlation against anharmonic motion effects is a new feature presented here.

## 2. Experimental and computational setup   

### 2.1. X-ray data collection   

**Figure 2:**
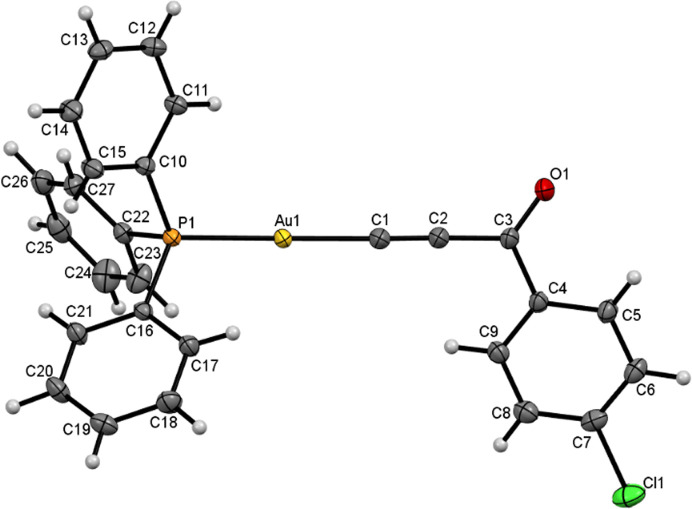
Molecular structure of the investigated gold(I) compound for the Ag *K*α data after IAM. The labeling scheme applies to all further refinements. Ellipsoids are drawn at the 50% probability level. Hydrogen atoms are shown as small spheres of arbitrary radius.

**Figure 3:**
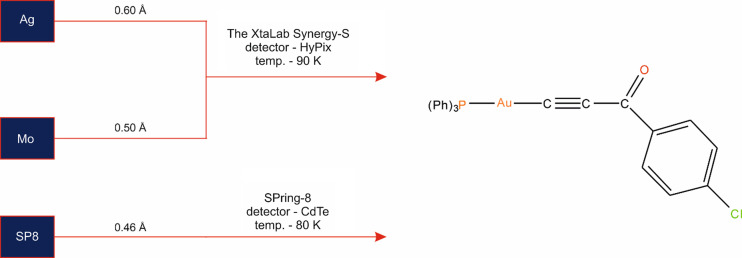
Scheme of X-ray experiments performed with applied resolution cut-offs.

Good-quality single crystals of the investigated compound (Fig. 2 ▸) were selected for high-resolution X-ray diffraction experiments using three different wavelengths (Fig. 3 ▸). Two diffraction datasets were collected on the XtaLAB Synergy-S instrument equipped with an HyPix-6000HE detector and a microfocus sealed tube source. The measurements were carried out using both Ag *K*α (λ = 0.56087 Å) and Mo *K*α (λ = 0.71073 Å) radiation at 90 and 93 K, respectively, hereafter referred to for simplicity as Ag and Mo data. The lattice parameters were obtained by least-squares fit to the optimized setting angles of the reflections collected using the *CrysAlis CCD* software (Rigaku Oxford Diffraction, 2015 ▸). Data were reduced using *CrysAlis RED* (Rigaku Oxford Diffraction, 2015 ▸). The face-based analytical absorption correction implemented in *CrysAlisPro* (Rigaku Oxford Diffraction, 2015 ▸) was applied to both datasets. High-resolution data were also collected using synchrotron radiation at the BL02B1 beamline of the SPring-8 synchrotron (SP8), Japan, with an X-ray energy of 50 keV (λ = 0.2486 Å) at a temperature of 80 K using a Huber 1/4χ-axis goniometer equipped with a Pilatus3 X 1M CdTe (P3) detector. The Pilatus images were converted to the Bruker .sfrm format using published software (Krause *et al.*, 2020 ▸) and integrated using *APEX3* (Bruker AXS Inc. Madison, WI, 2016 ▸). The multi-scan absorption correction was applied using *SADABS* (Sheldrick, 1996 ▸; Bruker AXS Inc. Madison, WI, 2016 ▸). The X-ray experimental details can be found in Table 1 ▸.

### 2.2. Structure determination   

Structure determination was carried out using *SHELX* (Sheldrick, 1990 ▸). The structure was solved with direct methods and then refinements were carried out based on full-matrix least-squares on *F* ^2^ using *SHELXL* (Sheldrick, 2016 ▸) for all measured data within the graphical interface of *Olex2* (Dolomanov *et al.*, 2009 ▸). We detected partial disorder of one phenyl ring, however, since *Tonto* it is unable to deal with disordered structures, we have decided to use an unmodeled component resulting in slightly larger carbon ADPs in this ring. The datasets obtained were subsequently scaled and merged using *SORTAV* (Blessing, 1995 ▸). The resulting models were used as a starting point for HAR, which was based on *F* and was performed against all reflections, except those with negative *F*. No I/σ cutoff was applied.

### 2.3. Hirshfeld atom refinement   

A series of HARs (Capelli *et al.*, 2014 ▸) were performed with *Tonto* (Grimwood *et al.*, 2003 ▸) (version: 20.04.15 v. 97c7857). The uncontracted cc-pVDZ basis set (Dunning, 1989 ▸) was used for all chemical elements with the exception of the Au atom, where the uncontracted DZP-DKH basis set (Barros *et al.*, 2010 ▸) was employed. SCF calculations were performed with a cluster of charges and dipoles in order to simulate the crystal environment of all neighboring molecules which have any atom within a radius of 8 Å from the central molecule. During HAR, all atomic positions were refined without any constraints or restraints. ADPs were refined only for C, P, O, Cl and Au, while H atoms were treated isotropically. Additionally, in some cases anharmonic thermal motions for the Au atom were refined up to fourth-order Gram–Charlier (GC) coefficients (Table 2 ▸). To explore the impact of relativistic effects, electron correlation and anharmonic thermal motions, HARs were performed at different levels of theory. Therefore, wavefunction calculations were run using restricted Hartree–Fock (rhf) and restricted Kohn–Sham (rks) methods. The rks calculations were carried out using the hybrid Becke-3-Lee-Yang-Parr (B3LYP) functional. The relativistic calculations were based on the IOTC Hamiltonian. The abbreviations of the refinements performed in this study with a description of the methods used and the effects observed are summarized in Table 2 ▸.

### 2.4. Anharmonic thermal motion analysis   

In order to confirm the presence of anharmonic nuclear motions, the probability density function (PDF) and the minimum data resolution required for meaningful refinement of the anharmonic displacement parameters (Kuhs, 1992 ▸) (Tables S8–S14 of the supporting information) were also analyzed, using *MoleCoolQT* (Hübschle & Dittrich, 2011 ▸) to visualize the PDFs and *XDPDF* (Volkov *et al.*, 2016 ▸) to estimate the resolution threshold.

## 3. Results and discussion   

### 3.1. Model quality   

The statistical parameters obtained from HAR at different levels of theory are listed in Tables 3 ▸ and S1–S3. The goodness-of-fit values range from 0.9 to 1.5 and are closer to unity when the anharmonic nuclear motions (third and fourth order) of the gold atom have been included (with the exception of the Ag data, for which the resolution was slightly below the minimum data resolution limit). A similar trend is also observed for the χ^2^ agreement statistics. Similarly to Bučinský *et. al.* (2016 ▸), rks_anh_rel HAR yielded better (closer to unity) agreement statistics compared with non-relativistic HAR (rks_anh_nr), which demonstrates that taking relativistic effects into account improves the reconstruction of electron density from the experiment (Tables 3 ▸ and S1–S3). The quality of the datasets collected and refinement models can also be confirmed by the values of the C—H bond lengths refined with HAR that agree quite well with the averaged value from neutron diffraction experiments of 1.08 Å (Allen & Bruno, 2010 ▸) (Tables S5–S7).

**Figure 4:**
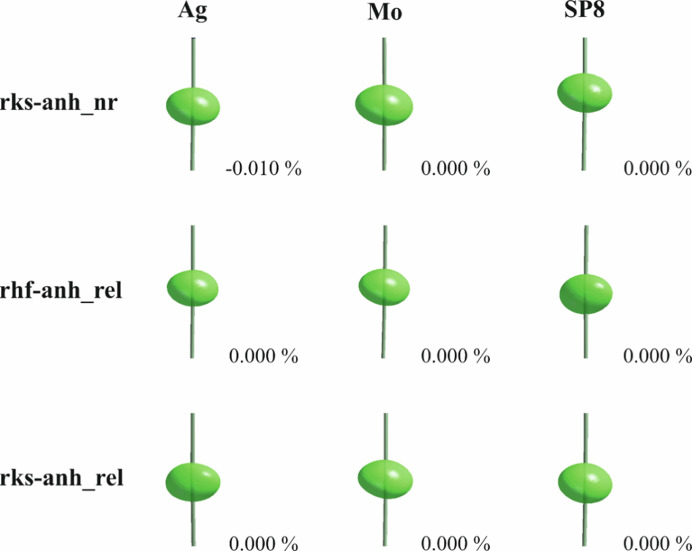
Graphical representation of the total probability density function of the gold atom at the 90% probability level for all anharmonic refinements considered. The percentage values denote total integrated negative probability.

The maximum positive and negative residual densities for the HARs with harmonic nuclear motions (rks_rel), when compared with IAM (Tables 1 ▸, 3 ▸ and S1–S3), became lower. However, a significant improvement is observed only in the case of the Ag data, which is at least in part due to the lower experimental resolution. Fractal dimension plots (for more information see the supporting information) (Meindl & Henn, 2008 ▸) for the rks_rel refinements are not narrow and deviations from the parabolic shape and pronounced shoulders can be observed for all three datasets (Figs. S8, S10 and S12 of the supporting information). Therefore, HARs including anharmonic nuclear motions of the gold atom up to third and fourth order of the Gram–Charlier coefficients were performed, which visibly reduced maximum positive and minimum negative residual densities (Tables 1 ▸, 3 ▸ and S1–S3; Figs. S7, S9 and S11). The minimum data resolution required for Au was achieved only in the case of the Mo and SP8 data for the refinement of third order of GC coefficients and was close to sufficient for the fourth order (Table S8). Of course, imperfections in the residual density maps can still be observed, but improvement seems to be significant in comparison to the rks_rel refinements [Figs. S7(*a*), S9(*a*) and S11(*a*)]. For all datasets, almost all GC coefficients were more significant than three standard uncertainties (Tables S9–S14). The derived total probability density functions for refinements with anharmonic nuclear motion of Au up to the fourth order showed only positive integrated probability and, therefore, no visible negative region around Au in the graphical representation (Fig. 4 ▸). These features indicate the presence of anharmonic vibrations and confirm their physical relevance.

### 3.2. Changes in dispalcement parameters   

In this subsection, we investigate changes in the ADPs of gold which arise from electron correlation and relativistic effects and compare changes in anharmonic displacement parameters for Ag, Mo and SP8 datasets. In Tables 4 ▸ and S15, we calculated the differences beween ADPs obtained from rks_nr, rhf_rel and rks_rel models for SP8, Ag and Mo data, respectively. The differences were calculated by subtracting rks_nr or rhf_rel from the rks_rel model. The graphical representation was generated using *Olex2* (Dolomanov *et al.*, 2009 ▸) (Fig. S4). Note that differences between ADPs are larger than three standard uncertainties only for diagonal *U*_11_, *U*_22_ and *U*_33_ elements of the ADP tensor and reflect systematic underestimation of ADPs by rks_nr and rhf_rel models, respectively. This means that the inclusion of electron correlation or relativistic effects leads to increased ADPs. The differences have isotropic shape for both ECORR and REL effects, however, they are smaller for the ECORR by an order of magnitude for SP8 data (Table 4 ▸). In general, these differences become larger with decreasing data resolution as follows: SP8 < Mo < Ag (Table S15).

**Figure 5:**
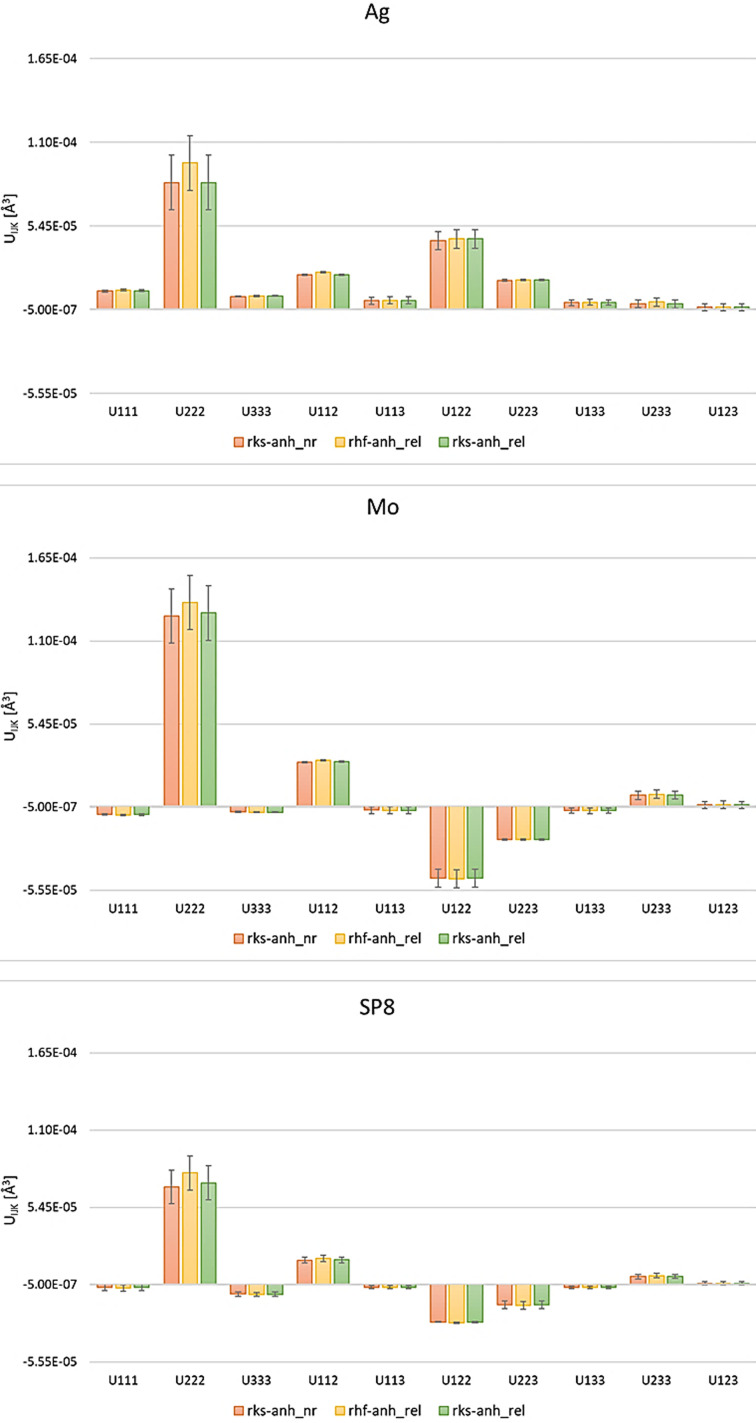
Plot of the numeric values of the significant third-order GC coefficients within three e.s.d.s for Ag, Mo and SP8 data for HARs at different levels of theory.

Figs. 5 ▸ and S2 show third and fourth order anharmonic tensor components with three estimated standard deviation (e.s.d.) values of these parameters obtained from refinements at different levels of theory for three datasets, respectively. The plots show that anharmonic displacement coefficients are independent of the method used in HAR. A comparison of the third-order GC coefficients clearly demonstrates that for the Ag data, the direction of some individual GC parameters is different from that for Mo or SP8 data (Fig. 5 ▸), thus indicating too-low data resolution of this particular dataset. Similar conclusions can be drawn when looking at the fourth-order GC coefficients (Fig. S2). Although the trend for the individual GC parameters is the same when comparing the Mo and SP8 data, the quantitative changes in the GC parameters for the Mo data are more similar to those for the Ag than for the SP8 data. The resulting features indicate that the resolution of the Mo data is slightly too low to refine the fourth order of anharmonic parameters, but their directions do not deviate from those obtained for the SP8 data, therefore we can consider this resolution as a borderline case.

### 3.3. Topological analysis of electron density   

To analyze the local impacts of various effects on the resulting electron density ρ(*r*), we employed topological analysis in the framework of the QTAIM (Bader, 1994 ▸). This allows for representation of the molecular structures in terms of molecular graphs and the corresponding bond paths, and it provides the atomic interaction characteristics in terms of ρ(*r*), ∇^2^ρ(*r*), the kinetic *G*(*r*) and potential *V*(*r*) energy densities as well as the local energy density *H*(*r*) at the BCPs.

In order to quantify the changes arising from the applied corrections, BCPs of the (3,−1) type were computed with the *Multiwfn* 3.8 (Lu & Chen, 2012 ▸) software. BCPs were found between all covalently bonded atoms as expected; however, only two BCPs for the Au—P and Au—C bonds will be discussed in detail. The most important topological characteristics of the two above mentioned bonds for all refinements considered are listed in Tables 5 ▸ and S16–S17.

#### 3.3.1. Relativistic effects   

At the geometry level, inclusion of relativistic effects yields no significant differences in the Au—P and Au—C bond distances (Table 5 ▸). This means that the relativistic change of the wavefunction has only a minor influence on bond distances, as reported in the literature (Snijders & Pyykkö, 1980 ▸). The BCP position is unchanged for the Au—C bond, but for Au—P the BCP is closer to the gold inner core when including relativistic effects. Changes arising from relativistic effects in topological properties of the electron density are clearly visible, and their significance increases in the order Ag < Mo < SP8 (Tables 5 ▸ and S16–17). Comparing ρ(*r*) at the BCPs of the above mentioned bonds, we find that the electron density increases on consideration of relativistic effects. The difference in ρ(*r*) between rks-anh_nr and rks-anh_rel refinements is larger for the Au—P than the Au—C bond with deviations of *ca* 5.2 and 2.7%, respectively (Table 5 ▸). Changes in the Laplacian of the electron density at the BCPs are even more detectable, since ∇^2^ρ(*r*) is a very sensitive quantity. Non-relativistic calculations (rks-anh_nr) result in a difference of 12.6 and 20.6% for Au—P and Au—C bonds, respectively, when compared with the rks-anh_rel refinements. The resulting differences in the energy densities suggest a slight stabilization of the investigated bonds on inclusion of the relativistic effects. The decrease in *H*_r_ is relatively small for the Au—P bond, however, it decreases rapidly for the Au—C bond (Table 5 ▸). Changes in the atomic charges are also observed. In general, inclusion of relativistic effects evidently decreases the charge of the heaviest element [Fig. S6(*a*)], whereas in case of the lighter atoms changes are barely observable (*e.g.* C1, C3, Cl1; Fig. S6).

**Figure 6:**
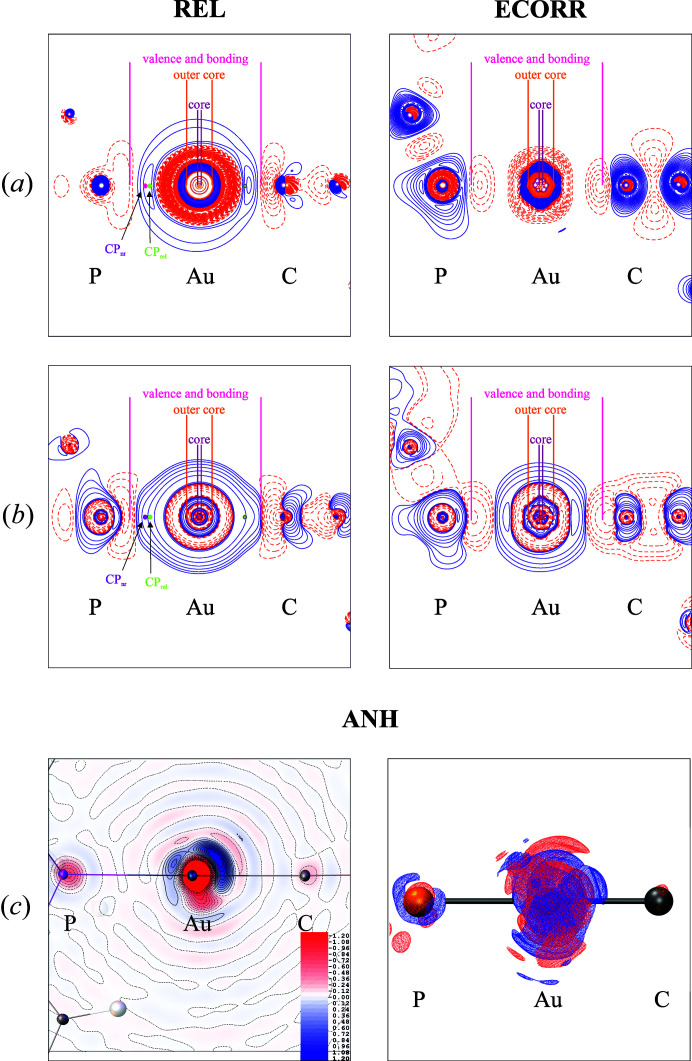
Difference maps for SP8 data of (*a*) static electron density (contour ±0.01 e A^−3^) and (*b*) negative Laplacian (contour values are in geometric order, starting from ±0.1 e A^−5^ with increments of 2 e A^−5^) exposing both the effects of relativity (REL) and electron correlation (ECORR). (*c*) 2D and 3D dynamic electron density in the plane of P—Au—C atoms exposing the effect of anharmonicity (ANH). Values of the positive and negative difference densities are denoted by blue solid and red dashed lines, respectively.

In Fig. 6 ▸, relativistic effects are shown as difference maps of the static electron density and the negative Laplacian obtained by subtracting the non-relativistic rks-anh_nr grid from the relativistic rks-anh_rel grid. As expected, the most significant difference in electron density is observed for the heavy element, although, even in the case of light atoms, a small influence of the relativistic effects is also visible [Fig. 6 ▸(*a*), left]. Electron density increases in BCPs on inclusion of relativistic effects as previously shown in Table 4 ▸. Difference maps of the negative Laplacian exhibit local charge depletion in the outer core of the metal atom and local charge concentration in the bonding region [the pink border lines, Fig. 6 ▸(*b*), left]. Both maps show that the distributions of the electron density further along the Au—P and Au—C bonds are different to each other as the electron density and Laplacian appear to be more reduced in the direction of the Au—C bond (Fig. 6 ▸).

#### 3.3.2. Electron correlation   

Inclusion of electron correlation within rks-anh_rel decreases ρ(*r*) and ∇^2^ρ(*r*) at the BCPs of both the Au—P and Au—C bonds (Table 5 ▸). Contrary to including relativistic effects, the change in the ρ(*r*) values for the Au—P bond is smaller with a deviation of only 1.3% (Table 5 ▸). For the Au—C bond, this change is almost as large as that caused by including relativity (*ca* 2.2%, Table 5 ▸). The resulting changes are again larger for ∇^2^ρ(*r*) than for ρ(*r*) and seem to be independent of the dataset for the Au—C bond (Tables 5 ▸ and S16–S17). However, this trend is not preserved for the Au–P bond. The resulting deviations are tremendous, and decrease slightly in the order Ag < Mo < SP8 (Tables 5 ▸ and S16–S17). When employing electron correlation for the Au—C bond the deviation is smaller than that caused by applying relativity (*ca* 10.7%, Table 5 ▸), but for the Au—P bond this deviation is dramatically higher with a value of 63.9% (Table 5 ▸). Changes in the energy densities on inclusion of the electron density for both bonds are very small; however, according to the Cremer and Kraka (1984*a* ▸,*b* ▸; Krawczuk & Macchi, 2014 ▸) classification, a slight destabilization of the Au—C bond is observed (Table 5 ▸). The rhf-anh_rel calculation underestimates or does not significantly change values of atomic charges, with the exception of the charges on the P1, C3, H5, H6, H8, H9, H13, H17, H20, H21 and H25–H27 atoms (Fig. S6).

The difference maps reveal that electron correlation dominates over the whole molecule [Fig. 6(*a*),(*b*), right ▸]. It is clear that electron correlation has a more global reach when compared with relativistic effects, which mostly dominate the area of the metal atom. Similarly, in the case of relativistic effects, inclusion of electron correlation is also involved with local charge depletion in the outer core region of the metal atom, which is visible in the difference maps [Fig. 6(*a*),(*b*), right ▸]. However, the behavior in the valence and bonding regions, when comparing ECORR and REL, is different, not only in the area of the gold atom, but also around the phospho­rous and carbon atoms. The local charge concentration in the valence region around the gold atom is more contracted and the local charge depletion is more elongated in the direction of the metal atom.

#### 3.3.3. Anharmonicity   

The introduction of anharmonic motion corrections for the gold atom produces very small changes in the topological parameters at the BCPs of the investigated bonds in Table 5 ▸, which can be attributed to small geometry differences after the refinements. The underlying quantum-chemical calculation of the static electron density is, however, identical in both models; the refined anharmonic motion parameters only influence the crystal dynamics. This feature is mostly seen in changes of residual electron density (Tables S6, S8 and S10). However, these changes are also visible in the dynamic electron density, which can be computed by inverse Fourier transformation of calculated structure factors. The manifestation of anharmonicity is visible in Fig. 6 ▸(*c*) (2D and 3D maps) as the difference between rks_anh_rel and rks_rel dynamic electron densities. The major effect of anharmonicity is found near the atomic position of gold and is most pronounced in the direction perpendicular to the Au—P or Au—C bonds [Fig. 6 ▸(*c*)], whereas no extrema are observed in the valence or bonding region.

### 3.4. Profiles of electron density   

**Figure 7:**
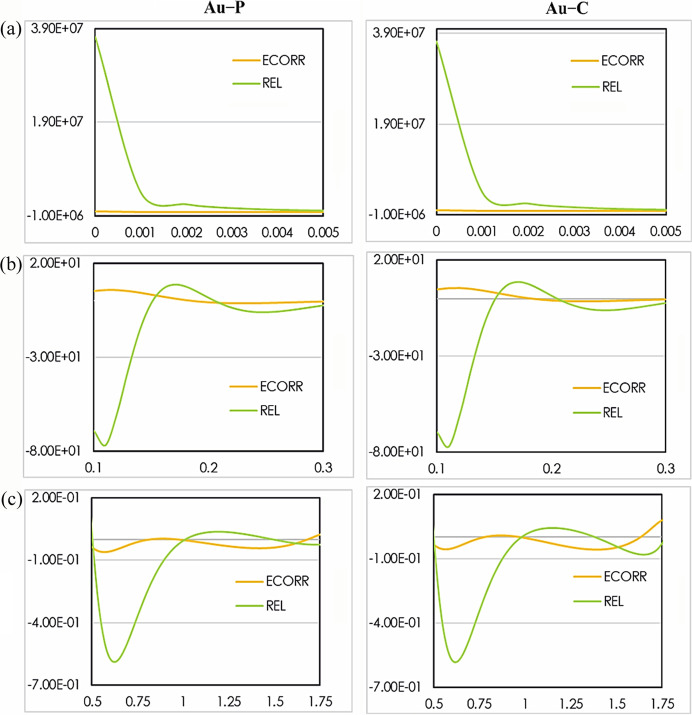
1D difference static electron density plots resulting from the relativistic and electron correlation effects (*y* axis, in eÅ^−3^) as a function of the Au—P and Au—C bond distance (*x* axis, in Å) of performed HARs against SP8 data.

The profiles of electron density along the Au—C and Au—P bonds for all considered refinements are presented in Figs. S13–S15 and represent global measures of the tested effects. In Fig. 7 ▸, we present difference static electron density plots resulting from HARs performed only against SP8 data for clarity. They show the relativistic and electron correlation effects. The plots exhibit the distribution of the electron density in the core (from 0.0 to 0.005 Å), outer core (from 0.1 to 0.3 Å), valence (from 0.5 to 1.75 Å) and bonding regions (from 1.2 to 1.5 Å) of the above mentioned bonds.

As it can be seen from Fig. 7 ▸, relativistic effects strongly dominate over the core region. In contrast, the effect of electron correlation in the core region is negligible. Relativistic effects remain most signifigant within the range 0.15–0.21 Å. This trend was also observed in different theoretical studies and has been already reported in the literature (Bučinský *et al.*, 2019 ▸; Gatti *et al.*, 2007 ▸). In the outer core region, electron correlation grows to become a key factor in the electron density behavior, too. A slight dominance of ECORR is noticeable in the range 0.2–0.3 Å. In the region from 0.5 to 1.0 Å, ECORR has the largest influence on the electron density, whereas in the bonding region (around 1.2 Å) relativistic effects tend to be the most important.

From Figs. 7 ▸ and S13−S15, it is clear that relativistic effects and electron correlation affect the distribution of the electron density along the Au—C and Au—P bonds. Moreover, the behavior of the electron density close to the gold inner core is very similar for Au—C and Au—P bonds. The values of the electron density at the gold inner core are in excellent agreement between all datasets, since differences in experimental geometries are very small. Comparison of the electron density values at the gold inner core also shows the importance of the application of electron correlation (rhf-anh_rel *versus* rks-anh_rel) and relativistic effects (rks-anh_nr *versus* rks-anh_rel), which are also visualized in Figs. S15(*a*) and S15(*b*). The non-relativistic curve (pink) lies at a lower level than all the relativisitic curves, which illustrates the well known phenomenon of relativistic contraction of electron density (Reiher, 2012 ▸; Reiher & Wolf, 2004 ▸; Dyall & Faegri, 2007 ▸) not readily seen in 2D maps.

### 3.5. Profiles of negative Laplacian   

**Figure 8:**
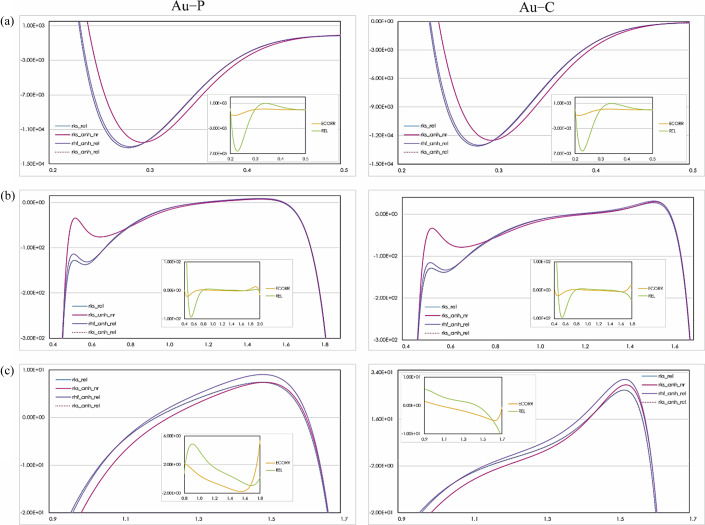
1D plots of negative Laplacian (*y* axis, in eÅ^−5^) as a function of the Au—P and Au—C bond distance (*x* axis, in Å) resulting from performed HARs against SP8 data. The subplots show difference electron densities resulting from the relativistic and electron correlation effects.

The distribution of the negative Laplacian of the electron densities along the Au—P and Au—C bonds for Ag, Mo and SP8 data are presented in Figs. 8 ▸, S12 and S13, respectively. The subplots show the influence of relativistic and electron correlation effects on the negative Laplacian profiles.

The first apparent difference between all refinements considered is a change in the positions of the minima of the non-relativistic and relativistic curves in the outer core region (from 0.2 to 0.3 Å). The electron depletion is shifted by 0.02 Å in the direction of the metal core, which represents a relativistic contraction. A significant difference between the magnitude of the local maxima in the region around 0.5 Å from Au [Figs. 8 ▸(*b*), S16(*b*) and S17(*b*)] is also detected. The non-relativistic curve (pink) always lies above all other curves which confirms the previously reported reduction of electron density concentration in this region due to relativity. However, this is only true for the metal atom, whereas the effect cannot be detected for lighter atoms [Fig. 8 ▸(*c*), S16(*c*) and S17(*c*)]. At this stage, it is worth pointing out that there is a further difference between charge depletion and concentration along the Au—P or Au—C bonds. We note that the maximum of the negative Laplacian profile around 1.5 Å indicates a local concentration of charge, whereas the outer core region of the Au atom is a region of local charge depletion, suggesting polarization of the Au—P and Au—C bonds towards the metal center.

For the above mentioned bonds, the shape and magnitude of the minima and maxima of the relativistic effects in the negative Laplacian profiles, which are present in the subplots, vary with the datasets analyzed (Figs. 8 ▸ and S16–S17). The most interesting changes, as expected, are observed in the nuclear region of the gold atom. In particular, the minimum of the non-relativistic curve (rks-anh_nr, pink line) lies above the minima of the relativistic curves (rks-anh_rel and rhf-anh_rel as dotted mulberry and solid violet lines, respectively) for all datasets [Figs. 8 ▸(*a*), S16(*a*) and S17(*a*)]. However, the magnitude of this minimum for the Ag data [subplots in Figs. S16(*a*) and S17(*a*)] deviates from the others by ∼340 e Å^−5^.

Moreover, in the region between 0.32 and 0.33 Å, the REL curve for the Ag data is not as flat as for the Mo or SP8 data (the magnitude of the local maximum is higher by about 100 e Å^−5^). In contrast, the magnitude of the electron correlation in the negative Laplacian remains the same for all datasets (subplots in Figs. 8 ▸, S16 and S17), however, it is significantly lower than for relativistic effects.

In summary, the negative Laplacian profiles of the models confirm the significance of the relativistic and electron correlation effects in the negative Laplacian distributions, especially at the Au inner core [region from 0.2 to 0.5 Å; subplots in Figs. 8 ▸(*a*), S12(*a*) and S13(*a*)]. This suggests that they can be detected experimentally for such heavy elements; however, in order to confirm this conclusion, a full X-ray wavefunction fitting procedure should be performed for the experimental X-ray dataset. Due to the partial disorder detected in the structure, the full X-ray wavefunction fitting procedure was not feasible because treatment of disordered structure is not possible in *Tonto* and the existing disorder might obscure the relativistic effects in the experimentally reconstructed electron density.

## 4. Conclusions and outlook   

In this work, we have successfully performed HAR with relativistic Hamiltonians for an organo-gold(I) compound. The quality of the models was significantly better for HAR than for IAM. When comparing the HAR models, the quality of the relativistic refinements proved to be higher than the non-relativistic refinements, indicated by the improved refinement statistics and flatter residual density maps. However, the most significant impact on the refinements resulted from the inclusion of anharmonic vibrations for the gold atom. We also showed that data resolution is the most important factor when an anharmonic model of thermal motion is applied (Fig. 4 ▸), even if several different criteria (Herbst-Irmer *et al.*, 2013 ▸; Krause *et al.*, 2017 ▸) such as visible reduction of residual density, a reasonable PDF or a more parabolic shape of fractal dimension plot are fulfilled.

We showed the impact of the relativistic and electron correlation effects on the theoretically calculated static electron densities, and the impact of atomic anharmonicity on the calculated dynamic electron density. The differences arising from the investigated effects in the electron density and negative Laplacian at the BCPs for Au–P or Au–C bonds are of significant magnitude. For both bonds, the electron density at the BCPs increases on inclusion of the relativistic effects, but decreases when electron correlation is accounted for (Table 5 ▸). Importantly, the effects of electron correlation on the topology of ρ(*r*) are comparable in magnitude to those found for relativity. The differences considered are much larger in the negative Laplacian at the BCPs, which demonstrates the usefulness of ∇^2^ρ in the detection of such subtle changes in electron density. These results are in good agreement with earlier studies (Eickerling *et al.*, 2007 ▸; Batke & Eickerling, 2016 ▸; Fischer *et al.*, 2011 ▸; Bučinský *et al.*, 2014 ▸, 2016 ▸).

The global measures of the investigated effects in the framework of difference maps showed that the electron correlation influences the whole charge distribution in contrast to relativistic effects and anharmonicity, which mostly dominate in the core area of the heavy atom. Nevertheless, electron density and negative Laplacian profiles demonstrate the significance of relativistic effects also in the Au—C/P bonding region (Figs. 7 ▸ and 8 ▸, the region around 1.2–1.5 Å along the Au—P and Au—C bonds).

Finally, by comparing the results for the Ag, Mo and SP8 datasets, we showed discrepancies between the Ag and Mo/SP8 models when analyzing the influence of relativistic effects. We noticed systematic changes in the topological properties of the electron density at the BCPs, arising from small differences between the final experimental geometries. To confirm that relativistic effects can be detected in experimentally reconstructed electron density, a full X-ray wavefunction fitting procedure should be performed. However, this challenging task will be published as a separate study.

When comparing the three datasets, we noticed that the results for the Ag data deviate from those obtained for the Mo and SP8 data by analysing both the relativistic effects and the anharmonicity. Within this observation, we can conclude that electron density studies of heavy elements require higher resolution data than 0.5 Å. Refinement of fourth-order Gram–Charlier coefficients requires even better quality data with even higher data resolution. This means that in-house laboratory sources cannot be disqualified for charge density studies of heavy elements, when measurements of really high-resolution data are possible. This usually requires fast single photon counting detectors since a long exposure time is not favorable in X-ray diffraction experiments for organic compounds with heavy elements. In this respect, synchrotron sources seem to be a very good alternative, but one has to be aware that this is not always the case. On synchrotrons, experiments are usually very fast and absorption of X-ray radiation is lower but, on the other hand, the risk of sample damage due to strong radiation is much higher and is very common. Therefore, there is no definite answer yet as to which source is better. Each organometallic sample is unique with its associated problems, therefore, each compound requires specific treatment.

We have shown that relativistic and electron correlation effects do influence electron density distribution in crystals of heavy metal compounds. Thus, analyses of properties, either magnetic or electronic, which are based on electron density studies could bring some wrong conclusions, when the description of the above mentioned effects is omitted. For example, fully relativistic calculations, together with gas-phase chromatography experiments, showed that flerovium (Fl) is not as inert as a ‘noble gas’, but is a ‘volatile metal’ (Pershina, 2011 ▸; Yakushev *et al.*, 2014 ▸). In other studies, it was found that the use of the nonrelativistic Lévy–Leblond Hamiltonian with the relativistic Dirac–Coulomb and spin-free hamiltonian allows separation of the scalar and spin-dependent relativistic contributions to the nuclear magnetic resonance (NMR) parameters (Romero, 2008 ▸). There are many other examples that highlight the importance of relativistic effects in different fields of studies (Rhodes & Semon, 2004 ▸; Aksenov *et al.*, 2017 ▸; Gates *et al.*, 2018 ▸; Epifano *et al.*, 2019 ▸; Pyper, 2020 ▸).

## 5. Related literature   

The following references are cited in the supporting information: Bronstein *et al.* (2008 ▸); Schwarzenbach *et al.* (1989 ▸).

## Supplementary Material

Crystal structure: contains datablock(s) mg14_IAM_Ag, mg14_ag_rhf_anh_rel, mg14_ag_rks_anh_nrel, mg14_ag_rks_anh_rel, mg14_ag_rks_rel, mg14-Mo_IAM, mg14_mo_rhf_anh_rel, mg14_mo_rks_anh_nrel, mg14_mo_rks_anh_rel, mg14_mo_rks_rel, SP8_IAM, mg14_spring8_rhf_anh_rel, mg14_spring8_rks_anh_nrel, mg14_SP8_rks_anh_rel, mg14_spring8_rks_rel. DOI: 10.1107/S2052252521004541/lt5037sup1.cif

Structure factors: contains datablock(s) mg14_Ag_IAM. DOI: 10.1107/S2052252521004541/lt5037sup2.fcf

Structure factors: contains datablock(s) mg14_ag_rhf_anh_rel. DOI: 10.1107/S2052252521004541/lt5037sup3.fcf

Structure factors: contains datablock(s) mg14_ag_rks_anh_nrel. DOI: 10.1107/S2052252521004541/lt5037sup4.fcf

Structure factors: contains datablock(s) mg14_ag_rks_anh_rel. DOI: 10.1107/S2052252521004541/lt5037sup5.fcf

Structure factors: contains datablock(s) mg14_ag_rks_rel. DOI: 10.1107/S2052252521004541/lt5037sup6.fcf

Structure factors: contains datablock(s) mg14_Mo_IAM. DOI: 10.1107/S2052252521004541/lt5037sup7.fcf

Structure factors: contains datablock(s) mg14_mo_rhf_anh_rel. DOI: 10.1107/S2052252521004541/lt5037sup8.fcf

Structure factors: contains datablock(s) mg14_mo_rks_anh_nrel. DOI: 10.1107/S2052252521004541/lt5037sup9.fcf

Structure factors: contains datablock(s) mg14_mo_rks_anh_rel. DOI: 10.1107/S2052252521004541/lt5037sup10.fcf

Structure factors: contains datablock(s) mg14_mo_rks_rel. DOI: 10.1107/S2052252521004541/lt5037sup11.fcf

Structure factors: contains datablock(s) SP8_IAM. DOI: 10.1107/S2052252521004541/lt5037sup12.fcf

Structure factors: contains datablock(s) mg14__SP8_rhf_anh_rel. DOI: 10.1107/S2052252521004541/lt5037sup13.fcf

Structure factors: contains datablock(s) mg14_SP8_rks_anh_nrel. DOI: 10.1107/S2052252521004541/lt5037sup14.fcf

Structure factors: contains datablock(s) mg14_SP8_rks_anh_rel. DOI: 10.1107/S2052252521004541/lt5037sup15.fcf

Structure factors: contains datablock(s) mg14_SP8_rks_rel. DOI: 10.1107/S2052252521004541/lt5037sup16.fcf

Supporting figures and tables. DOI: 10.1107/S2052252521004541/lt5037sup17.pdf

CCDC references: 2043573, 2043574, 2043575, 2043576, 2043577, 2043578, 2043579, 2043580, 2043581, 2043582, 2043583, 2043584, 2043585, 2043586, 2043587

## Figures and Tables

**Table 1:** X-ray data collection and structure refinement details of the Ag, Mo and SP8 datasets Final *R* indices are provided for the IAM model refined in *SHEXL* (Sheldrick, 2016 ▸).

		Ag		Mo		SP8
Empirical formula		C_27_H_19_AuClOP		C_27_H_19_AuClOP		C_27_H_19_AuClOP
Formula weight (g mol^−1^)		622.81		622.81		622.81
Crystal system		Monoclinic		Monoclinic		Monoclinic
Space group		*C*2/*c*		*C*2/*c*		*C*2/*c*
*Z*		8		8		8
*F*(000)		2400.0		2400.0		2400.0
Radiation (Å)		Ag *K*α (λ = 0.56087)		Mo *K*α (λ = 0.71073)		Synchrotron (λ = 0.2482)
*a* (Å)		17.6904 (2)		17.6896 (2)		17.7234 (6)
*b* (Å)		12.22917 (16)		12.2436 (1)		12.2442 (5)
*c* (Å)		21.2808 (2)		21.2660 (7)		21.3184 (8)
β (°)		94.6132 (10)		94.6500 (9)		94.6480 (16)
Volume (Å^3^)		4588.96 (9)		4590.69 (8)		4611.1 (3)
Temperature (K)		90.00 (15)		93.0 (3)		80
Absorption correction		Analytical		Analytical		Multi-scan
*T*_min_/*T*_max_		0.592/0.689		0.379/0.585		0.663/0.744
ρ_calc_ (g cm^−3^)		1.803		1.802		1.794
μ (mm^−1^)		3.605		6.612		0.377
Crystal size (mm)		0.19 × 0.136 × 0.111		0.2 × 0.139 × 0.11		0.08 × 0.06 × 0.09
2θ range for data collection (°)		4.304 to 55.728		3.844 to 90.588		1.414 to 30.99
Index ranges		−29 ≤ *h* ≤ 29		−35 ≤ *h* ≤ 35		−38 ≤ *h* ≤ 38
		−20 ≤ *k* ≤ 20		−24 ≤ *k* ≤ 24		−26 ≤ *k* ≤ 26
		−35 ≤ *l* ≤ 35		−42 ≤ *l* ≤ 42		−45 ≤ *l* ≤ 45
Reflections collected		66469		482446		288963
Independent reflections		11101		19253		237430
*R*_int_		0.0237		0.0539		0.0582
*R*_sigma_		0.0197		0.0107		0.0221
Data, restraints, parameters		11101, 0, 356		19253, 0, 280		23743, 0, 280
Goodness-of-fit on *F* ^2^		1.109		1.020		1.086
Final *R* indices [*I* ≥ 2σ (*I*)]		*R*_1_ = 0.0156, *w* *R*_2_ = 0.0375		*R*_1_ = 0.0173, *w* *R*_2_ = 0.0439		*R*_1_ = 0.0179, *w* *R*_2_ = 0.0454
Final *R* indices (all data)		*R*_1_ = 0.0184, *w* *R*_2_ = 0.0391		*R*_1_ = 0.0234, *w* *R*_2_ = 0.0456		*R*_1_ = 0.0217, *w* *R*_2_ = 0.0499
Largest diffraction peak/hole (eÅ^−3^)		1.29/−0.69		2.76/−0.44		2.06/−1.10

**Table 2:** Abbreviations of performed refinements

Abbreviations	Method used	Effects included
rks-anh_nr	Non-relativistic rks/B3LYP with anharmonic nuclear motions of Au	Electron correlation, anharmonicity
rks_rel	Relativistic rks/B3LYP with harmonic nuclear motions	Relativistic effects, electron correlation
rhf-anh_rel	Relativistic rhf with anharmonic nuclear motions of Au	Relativistic effects, anharmonicity
rks-anh_rel	Relativistic rks/B3LYP with anharmonic nuclear motions of Au	Relativistic effects, electron correlation, anharmonicity
		
Abbreviations	Difference between refinements	Effect observed
REL	rks-anh_rel – rks-anh_nr	Relativistic effects
ECORR	rks-anh_rel – rhf-anh_rel	Electron correlation
ANH	rks-anh_rel – rks_rel	Anharmonicity

**Table 3:** Statistical parameters of all HARs considered for the Ag, Mo and synchrotron data

	Ag data			
	rks-anh_nr	rks_rel	rhf-anh_rel	rks-anh_rel
*R*(*F*) (%)	1.59	1.71	1.57	1.59
*w* *R*(*F*) (%)	1.78	1.90	1.76	1.78
χ^2^	0.965	1.099	0.951	0.970
GooF	0.983	1.048	0.975	0.980
ρ_max_, ρ_min_ (eÅ^−3^)	0.63, −0.58	1.66, −0.62	0.63, −0.54	0.63, −0.58
Data, restraints, parameters	11101, 0, 381	11102, 0, 356	11102, 0, 381	11101, 0, 381
				
	Mo data			
	rks-anh_nr	rks_rel	rhf-anh_rel	rks-anh_rel
*R*(*F*) (%)	1.91	2.37	1.91	1.92
*w* *R*(*F*) (%)	2.01	2.41	2.01	2.01
χ^2^	1.505	2.157	1.494	1.500
GooF	1.227	1.469	1.222	1.220
ρ_max_, ρ_min_ (eÅ^−3^)	0.98, −0.89	3.54, −0.97	1.01, −1.18	1.06, −0.97
Data, restraints, parameters	19255, 0, 381	19255, 0, 356	19255, 0, 381	19255, 0, 381
				
	SP8 data			
	rks-anh_nr	rks_rel	rhf-anh_rel	rks-anh_rel
*R*(*F*) (%)	1.80	2.00	1.79	1.80
*w* *R*(*F*) (%)	2.78	2.94	2.78	2.78
χ^2^	1.766	1.974	1.761	1.760
GooF	1.329	1.405	1.327	1.330
ρ_max_, ρ_min_ (eÅ^−3^)	1.13, −0.88	3.30, −1.01	1.14, −0.82	1.20, −0.84
Data, restraints, parameters	23104, 0, 381	23104, 0, 356	23103, 0, 381	23102, 0, 381

**Table 4:** The difference between gold ADPs (Å^2^) obtained from rks_nr, rhf_rel and rks_rel, representing the effects of electron correlation (rks_rel – rhf_rel) and relativity (rks_rel – rks_nr) for SP8 data

Au	rks_nr	rhf_rel	rks_rel	ECORR	REL
*U*_11_	0.01634 (1)	0.01638 (1)	0.01647 (1)	0.00009	0.00013
*U*_22_	0.02160 (2)	0.02163 (2)	0.02173 (2)	0.00009	0.00013
*U*_33_	0.01284 (1)	0.01289 (1)	0.01297 (1)	0.00008	0.00013
*U*_12_	−0.003551 (7)	−0.003553 (7)	−0.003549 (7)	0.00000	0.00000
*U*_13_	0.001729 (8)	0.001727 (8)	0.001736 (8)	0.00001	0.00001
*U*_23_	−0.002987 (7)	−0.002990 (7)	−0.002985 (7)	0.00001	0.00000

**Table 5:** Selected BCP topological properties of Au—C and Au—P bonds resulting from wavefunction analysis obtained with HARs for SP8 data "dev" represents changes in the ρ(*r*) and ∇^2^ρ(*r*) values arising from REL, ECORR and ANH and are expressed in percentages relative to the rks-anh_rel values.

	Au—C								
	*r*_Au-BCP_ (Å)	*r*_Au—C_ (Å)	ρ(*r*) (eÅ^−3^)	dev (%)	∇^2^ρ(*r*) (eÅ^−5^)	dev (%)	*V*_r_ (Haa_0_^−3^)	*G*_r_ (Haa_0_^−3^)	*H*_r_ (Haa_0_^−3^)
rks-anh_nr	1.07	1.9892 (7)	0.925	−2.7	9.005	20.6	−0.1964	0.1449	−0.0515
rhf-anh_rel	1.06	1.9887 (7)	0.972	2.2	8.279	10.9	−0.2131	0.1495	−0.0636
rks-rel	1.07	1.9890 (8)	0.950	−0.11	7.462	−0.27	−0.1960	0.1367	−0.0593
rks-anh_rel	1.07	1.9887 (7)	0.951		7.464		−0.1962	0.1368	−0.0594
									
	Au—P								
	*r*_Au-BCP_ (Å)	*r*_Au—P_ (Å)	ρ(*r*) (eÅ^−3^)	dev %	∇^2^ρ(*r*) (eÅ^−5^)	dev %	*V*_r_ (Haa_0_^−3^)	*G*_r_ (Haa_0_^−3^)	*H*_r_ (Haa_0_^−3^)
rks-anh_nr	1.20	2.2773 (2)	0.733	−5.2	0.811	−12.6	−0.1163	0.0624	−0.0540
rhf-anh_rel	1.16	2.2772 (2)	0.783	1.3	1.521	63.9	−0.1370	0.0801	−0.0569
rks-rel	1.17	2.2767 (2)	0.773	0.0	0.926	−0.22	−0.1240	0.0667	−0.0572
rks-anh_rel	1.17	2.2773 (2)	0.773		0.928		−0.1238	0.0667	−0.0571
