# Supplementary material for: Relativistic Hirshfeld atom refinement of an organo-gold(I) compound
Source: IUCrJ. 2021 May 26;8(Pt 4):608–20. doi: 10.1107/S2052252521004541 (PMC8256711; doi:10.1107/S2052252521004541)
Supplement: Supplementary file 17 [file m-08-00608-sup17.pdf]

IUCrJ

Volume 8 (2021)

Supporting information for article:

Relativistic Hirshfeld atom refinement of an organo-gold(I) compound

Sylwia Pawlędzio, Maura Malinska, Magdalena Woińska, Jakub Wojciechowski, Lorraine Andrade Malaspina, Florian Kleemiss, Simon Grabowsky and Krzysztof Woźniak

S1. Meindl-Henn fractal dimensions plots

For good quality data, a Gaussian distribution (Schwarzenbach *et al.*, 1989) of the residual electron density in the unit cell is expected. The most common method used in crystallography, which describes distribution of the residual density is the so-called Meindl-Henn fractal dimension plot. In this technique, the dimension of the residual density isosurface of constant value is defined by a method called the box-counting dimension or Hausdorff dimension d^f, and is defined according to Bronstein *et al.* (Bronstein *et al.*, 2008):

$$d^f(\rho_0) = x = \lim_{\varepsilon \to 0} \frac{\log N(x,\ \varepsilon)}{\log N\ (\frac{1}{\varepsilon})}$$

where ε is the length of a box covering the unit cell under consideration, and N(x, ε) is the number of boxes which contain unit cell. For a theoretical data, where no noise is present and model describes electron density ideally ($F_{obs} = F_{calc}$), the isosurface of $\rho_0 = 0$ eÅ^{-3} fill unit cell completely with a d^f equal to 3. If only noise is present in the data, but no model errors are present, positive and negative residual density appears. In this case, the shape of the fractal dimension plot is parabolic. The upper limit of the d^f at $\rho_0 = 0$ eÅ^{-3} decreases to a non-integer value, which is characteristic for any experimental case. The value of d^f at $\rho_0 = 0$ eÅ^{-3} indicates featurelessness (the higher value, the less features) in the distribution of residual density. The difference between the values of maximum and minimum residual density corresponds to the flatness of the distribution of residual density (the lower difference, the flatter distribution).

References:

Schwarzenbach, D., Abrahams, S. C., Flack, H. D., Gonschorek, W., Hahn, T., Huml, K., Marsh, R. E., Prince, E., Robertson, B. E., Rollett, J. S. & Wilson, A. J. C. (1989). *Acta Cryst A*. **45**, 63–75.

Bronstein, I. N., Semendjajew, K. A., Musiol, G. & Muehlig, H. (2008). Taschenbuch der Mathematik Frankfurt am Main: Deutsch.

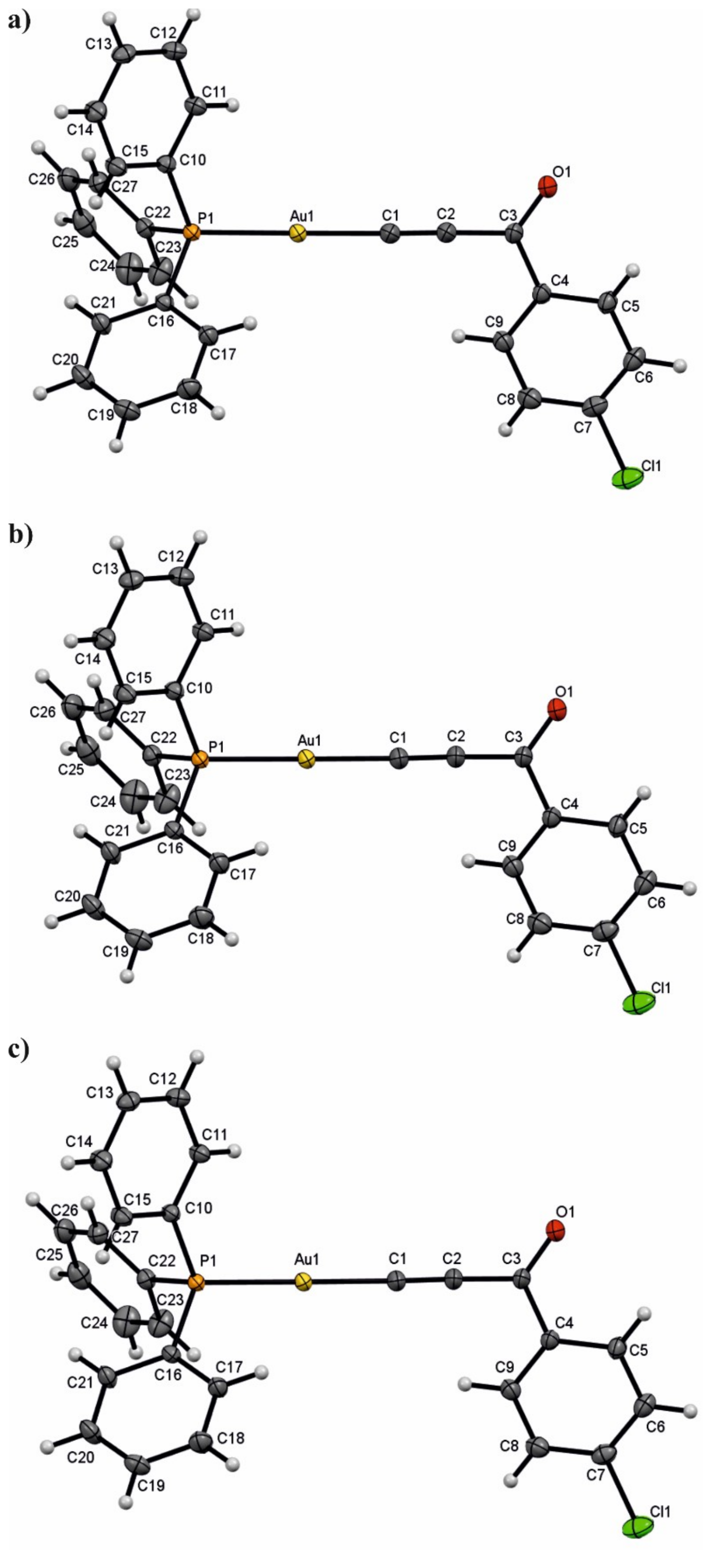

Figure S1 ORTEP-style representation of the molecule for the (a) Ag, (b) Mo and (c) SP8 data after IAM with labelling scheme. Ellipsoids are drawn at 50% probability level. H atoms are shown as small spheres of arbitrary radius.

Table S1 Statistical parameters of the HARs for the Ag data.

	rks-anh_nr	rks_rel	rhf-anh_rel	rks-anh_rel
**R_sig(F)**	2.72%	2.72%	2.72%	2.72%
**R_sig(F^2)**	0.11%	0.11%	0.11%	0.11%
**R(F)**	1.59%	1.71%	1.57%	1.59%
**R(F^2)**	1.49%	1.73%	1.46%	1.50%
***w*R (F)**	1.78%	1.90%	1.76%	1.78%
***w*R (F^2)**	NaN	NaN	NaN	NaN
**N_r**	11101	11101	11102	11101
**N_p**	381	356	381	381
χ^2	0.965	1.101	0.951	0.970
**GooF**	0.983	1.049	0.975	0.980
**Effective (mean) σ^2**	0.0835	0.0790	0.0840	0.0836
**Scale**	0.1226	0.1214	0.1229	0.1225
ρ_{max}	0.63	1.66	0.63	0.63
ρ_{min}	-0.58	-0.62	-0.54	-0.58

Table S2 Statistical parameters of the HARs for the Mo data.

	**rks-anh_nr**	**rks_rel**	**rhf-anh_rel**	**rks-anh_rel**
**R_sig(F)**	3.17%	3.17%	3.17%	3.17%
**R_sig(F^2)**	0.14%	0.14%	0.14%	0.14%
**R(F)**	1.91%	2.31%	1.91%	1.92%
**R(F^2)**	1.55%	2.62%	1.56%	1.59%
***w*R (F)**	2.01%	2.33%	2.01%	2.01%
***w*R (F^2)**	NaN	NaN	NaN	NaN
**N_r**	19255	19255	19255	19255
**N_p**	381	356	381	381
χ^2	1.506	2.019	1.494	1.500
**GooF**	1.227	1.421	1.222	1.220
**Effective (mean) σ^2**	0.0336	0.0364	0.0339	0.0341
**Scale**	0.1231	0.1197	0.1231	0.1227
ρ_{max}	0.98	3.54	1.01	1.06
ρ_{min}	-0.89	-0.97	-1.18	-0.97

Table S3 Statistical parameters of the HARs for the SP8 data.

	rks-anh_nr	rks_rel	rhf-anh_rel	rks-anh_rel
**R_sig(F)**	2.52%	2.52%	2.52%	2.52%
**R_sig(F^2)**	0.04%	0.04%	0.04%	0.04%
**R(F)**	1.80%	1.96%	1.79%	1.80%
**R(F^2)**	2.33%	2.73%	2.33%	2.38%
***w*R (F)**	2.78%	2.91%	2.78%	2.78%
***w*R (F^2)**	NaN	216.72%	NaN	NaN
**N_r**	23104	23102	23103	23102
**N_p**	381	356	381	381
χ^2	1.766	1.927	1.761	1.760
**GooF**	1.329	1.388	1.327	1.330
**Effective (mean) σ^2**	0.0694	0.0675	0.0694	0.0699
**Scale**	0.1224	0.1210	0.1223	0.1220
ρ_{max}	1.13	3.30	1.14	1.20
ρ_{min}	-0.88	-1.01	-0.82	-0.84

Table S4 Bond lengths of the Au-P and Au-C after HARs.

	Ag		Mo		Synchrotron	
	**Au1-P1**	**Au1-C1**	**Au1-P1**	**Au1-C1**	**Au1-P1**	**Au1-C1**
**rks-anh_nr**	2.2742(2)	1.9896(10)	2.27255(18)	1.9854(7)	2.27732(19)	1.9892(7)
**rhf-anh_rel**	2.2741(2)	1.9886(10)	2.27255(18)	1.9856(7)	2.27718(19)	1.9887(7)
**rks_rel**	2.2733(3)	1.9892(10)	2.2725(2)	1.9851(8)	2.27674(19)	1.9890(8)
**rks-anh_rel**	2.2742(2)	1.9884(10)	2.27255(18)	1.9854(7)	2.27726(19)	1.9887(7)

Table S5 C–H bond lengths after HARs for the Ag data.

	**rks-anh_nr**	**rhf-anh_rel**	**rks_rel**	**rks-anh_rel**
C5–H5	1.14(2)	1.14(2)	1.13(2)	1.14(2)
C6–H6	1.09(2)	1.09(1)	1.14(1)	1.09(1)
C8–H8	1.06(2)	1.06(2)	1.06(2)	1.06(2)
C9–H9	0.99(2)	0.97(2)	1.07(2)	0.98(2)
C11–H11	1.08(2)	1.08(2)	1.10(2)	1.08(2)
C12–H12	1.09(2)	1.09(2)	1.05(2)	1.09(2)
C13–H13	1.03(2)	1.01(2)	0.98(2)	1.03(2)
C14–H14	1.00(2)	1.00(2)	1.02(2)	1.00(2)
C15–H15	1.08(2)	1.08(2)	1.09(2)	1.08(2)
C17–H17	1.04(2)	1.04(2)	1.04(2)	1.04(2)
C18–H18	1.15(2)	1.14(2)	1.13(2)	1.14(2)
C19–H19	1.11(2)	1.12(2)	1.07(2)	1.11(2)
C20–H20	1.12(2)	1.13(2)	1.10(2)	1.12(2)
C21–H21	1.11(2)	1.11(2)	1.15(2)	1.11(2)
C23–H23	1.11(2)	1.11(2)	1.19(2)	1.11(2)
C24–H24	1.21(2)	1.20(2)	1.19(2)	1.21(2)
C25–H25	1.15(2)	1.14(2)	1.18(2)	1.15(2)
C26–H26	1.12(2)	1.12(2)	1.16(2)	1.12(2)
C27–H27	1.14(2)	1.12(2)	1.15(2)	1.14(2)
**C_{avr}**	1.09(2)	1.09(2)	1.11(2)	1.09(2)

Table S6 C–H bond lengths after HARs for the Mo data.

	**rks-anh_nr**	**rhf-anh_rel**	**rks_rel**	**rks-anh_rel**
C5–H5	1.12(2)	1.12(2)	1.13(2)	1.12(2)
C6–H6	1.12(2)	1.13(2)	1.12(2)	1.12(2)
C8–H8	1.07(2)	1.08(2)	1.08(2)	1.07(2)
C9–H9	1.08(2)	1.08(2)	1.07(2)	1.08(2)
C11–H11	1.14(2)	1.14(2)	1.14(2)	1.14(2)
C12–H12	1.05(2)	1.04(2)	1.03(2)	1.05(2)
C13–H13	1.11(2)	1.12(2)	1.11(3)	1.11(2)
C14–H14	1.03(2)	1.03(2)	1.04(3)	1.03(2)
C15–H15	1.07(2)	1.07(2)	1.06(2)	1.07(2)
C17–H17	1.04(2)	1.05(2)	1.03(2)	1.04(2)
C18–H18	1.09(3)	1.08(3)	1.08(2)	1.09(3)
C19–H19	1.11(2)	1.11(2)	1.11(2)	1.11(2)
C20–H20	1.08(2)	1.09(2)	1.10(2)	1.08(2)
C21–H21	1.18(2)	1.18(2)	1.18(2)	1.18(2)
C23–H23	1.08(2)	1.07(2)	1.07(3)	1.08(2)
C24–H24	1.17(2)	1.17(2)	1.14(4)	1.17(3)
C25–H25	1.09(2)	1.09(2)	1.07(2)	1.09(2)
C26–H26	1.11(3)	1.12(3)	1.10(3)	1.11(2)
C27–H27	1.11(2)	1.11(2)	1.08(3)	1.11(2)
**C_{avr}**	1.10(2)	1.10(2)	1.09(2)	1.10(2)

Table S7 C–H bond lengths after HARs for the SP8 data.

	rks-anh_nr	rhf-anh_rel	rks_rel	rks-anh_rel
C5–H5	1.09(2)	1.09(2)	1.10(2)	1.09(2)
C6–H6	1.08(2)	1.10(2)	1.09(3)	1.09(2)
C8–H8	1.04(2)	1.05(2)	1.11(2)	1.05(2)
C9–H9	1.10(2)	1.11(2)	1.10(2)	1.10(2)
C11–H11	1.10(2)	1.10(2)	1.09(3)	1.10(2)
C12–H12	1.08(2)	1.09(2)	1.10(2)	1.08(2)
C13–H13	1.03(2)	1.04(2)	1.12(2)	1.03(2)
C14–H14	1.07(2)	1.07(2)	1.16(3)	1.07(2)
C15–H15	1.08(2)	1.09(2)	1.13(3)	1.08(2)
C17–H17	1.06(2)	1.07(2)	1.09(2)	1.06(2)
C18–H18	1.08(3)	1.08(3)	1.08(2)	1.08(3)
C19–H19	1.10(2)	1.10(2)	1.02(2)	1.10(2)
C20–H20	1.08(2)	1.10(2)	1.08(2)	1.09(2)
C21–H21	1.12(2)	1.13(2)	1.05(2)	1.13(2)
C23–H23	1.08(2)	1.08(2)	1.07(2)	1.08(2)
C24–H24	1.10(2)	1.10(2)	1.06(2)	1.10(2)
C25–H25	1.16(2)	1.16(2)	1.06(3)	1.16(2)
C26–H26	1.12(3)	1.12(3)	1.08(2)	1.13(3)
C27–H27	1.11(2)	1.12(2)	1.09(2)	1.11(2)
**C_{avr}**	1.09(2)	1.09(2)	1.09(2)	1.09(2)

Table S8 The minimum data resolution required (in Å^{-1}), according to the Kuhs equation (ref) and numbers of values of Gram–Charlier coefficients larger than 3 σ for anharmonic refinements.

			**Ag data**			
	**rks-anh_nr**		**rhf-anh_rel**		**rks-anh_rel**	
	**res.**	**par.**	**res.**	**par.**	**res.**	**par.**
**exp.**	0.83		0.83		0.83	
**3rd**	0.99	9	0.98	9	0.99	9
**4th**	1.14	13	1.13	12	1.14	13
			**Mo data**			
	**rks-anh_nr**		**rhf-anh_rel**		**rks-anh_rel**	
	**res.**	**par.**	**res.**	**par.**	**res.**	**par.**
**exp.**	1.00		1.00		1.00	
**3rd**	0.94	9	0.93	9	0.93	9
**4th**	1.08	15	1.07	15	1.08	15
	**SP8 data**					
	**rks-anh_nr**		**rhf-anh_rel**		**rks-anh_rel**	
	**res.**	**par.**	**res.**	**par.**	**res.**	**par.**
**exp.**	1.11		1.11		1.11	
**3rd**	1.01	9	1.00	9	1.01	9
**4th**	1.17	15	1.16	15	1.16	15

Table S9 3rd order of the Gram-Charlier coefficients obtained for the Ag data. Values higher than three standard uncertainties are highlighted in orange.

	rks-anh_nr	rhf-anh_rel	rks-anh_rel
U_{111}	0.0000117(18)	0.0000125(19)	0.0000121(19)
U_{222}	0.000083(6)	0.000096(6)	0.000083(6)
U_{333}	0.0000083(10)	0.0000086(10)	0.0000087(10)
U_{112}	0.0000226(15)	0.0000240(15)	0.0000225(15)
U_{113}	0.0000054(8)	0.0000057(8)	0.0000057(8)
U_{122}	0.000045(2)	0.000046(2)	0.000046(2)
U_{223}	0.0000188(17)	0.0000191(17)	0.0000191(17)
U_{133}	0.0000041(6)	0.0000044(6)	0.0000043(6)
U_{233}	0.0000034(9)	0.0000045(9)	0.0000033(9)
U_{123}	0.0000012(8)	0.0000012(8)	0.0000012(8)

Table S10 4th order of the Gram-Charlier coefficients obtained for the Ag data. Values higher than three standard uncertainties are highlighted in orange.

	rks-anh_nr	rhf-anh_rel	rks-anh_rel
U_{1111}	0.0000036(10)	0.0000094(10)	0.0000076(10)
U_{2222}	0.000061(5)	0.000089(5)	0.000079(5)
U_{3333}	0.0000020(5)	0.0000047(5)	0.0000040(5)
U_{1112}	0.0000011(6)	0.0000007(6)	0.0000007(6)
U_{1113}	0.0000030(3)	0.0000037(4)	0.0000036(4)
U_{1222}	0.0000070(13)	0.0000064(13)	0.0000062(13)
U_{2223}	0.0000035(9)	0.0000026(10)	0.0000023(10)
U_{1333}	0.0000010(2)	0.0000015(2)	0.0000015(2)
U_{2333}	0.0000018(3)	0.0000016(3)	0.0000015(3)
U_{1122}	0.0000068(8)	0.0000113(8)	0.0000097(8)
U_{1133}	0.0000001(2)	0.0000016(2)	0.0000012(2)
U_{2233}	0.0000035(5)	0.0000064(5)	0.0000055(5)
U_{1123}	0.0000008(2)	0.0000005(3)	0.0000005(3)
U_{1223}	0.0000043(4)	0.0000048(4)	0.0000048(4)
U_{1233}	0.0000007(2)	0.0000004(2)	0.0000005(2)

Table S11 3rd order of the Gram-Charlier coefficients obtained for the Mo data. Values higher than three standard uncertainties are highlighted in orange.

	rks-anh_nr	rhf-anh_rel	rks-anh_rel
U_{111}	-0.0000053(11)	-0.0000057(11)	-0.0000055(11)
U_{222}	0.000126(4)	0.000135(4)	0.000128(4)
U_{333}	-0.0000037(6)	-0.0000039(6)	-0.0000039(6)
U_{112}	0.0000291(8)	0.0000305(8)	0.0000295(8)
U_{113}	-0.0000025(4)	-0.0000027(4)	-0.0000026(4)
U_{122}	-0.0000475(13)	-0.0000479(13)	-0.0000477(13)
U_{223}	-0.0000219(10)	-0.0000220(10)	-0.0000219(10)
U_{133}	-0.0000027(4)	-0.0000029(4)	-0.0000028(4)
U_{233}	0.0000073(5)	0.0000081(5)	0.0000075(5)
U_{123}	0.0000010(4)	0.0000011(4)	0.0000010(4)

Table S12 4th order of the Gram-Charlier coefficients obtained for the Mo data. Values higher than three standard uncertainties are highlighted in orange.

	rks-anh_nr	rhf-anh_rel	rks-anh_rel
U_{1111}	0.0000125(5)	0.0000141(5)	0.0000131(5)
U_{2222}	0.000066(2)	0.000076(3)	0.000070(2)
U_{3333}	0.0000077(2)	0.0000084(2)	0.0000079(2)
U_{1112}	-0.0000020(3)	-0.0000020(3)	-0.0000018(3)
U_{1113}	0.00000304(17)	0.00000324(17)	0.00000319(17)
U_{1222}	-0.0000076(7)	-0.0000077(7)	-0.0000073(7)
U_{2223}	-0.0000060(5)	-0.0000057(5)	-0.0000056(5)
U_{1333}	0.00000159(12)	0.00000173(12)	0.00000170(12)
U_{2333}	-0.00000078(15)	-0.00000067(15)	-0.00000062(15)
U_{1122}	0.0000094(3)	0.0000108(4)	0.0000100(4)
U_{1133}	0.00000316(11)	0.00000355(11)	0.00000331(11)
U_{2233}	0.0000062(2)	0.0000071(2)	0.0000065(2)
U_{1123}	-0.00000072(12)	-0.00000059(12)	-0.00000060(12)
U_{1223}	0.00000315(18)	0.00000330(18)	0.00000325(18)
U_{1233}	-0.00000061(10)	-0.00000050(10)	-0.00000051(10)

Table S13 3rd order of the Gram-Charlier coefficients obtained for the SP8 data. Values higher than three standard uncertainties are highlighted in orange.

	rks-anh_nr	rhf-anh_rel	rks-anh_rel
U_{111}	-0.0000023(8)	-0.0000029(8)	-0.0000025(8)
U_{222}	0.000069(4)	0.000079(4)	0.000072(4)
U_{333}	-0.0000072(5)	-0.0000074(5)	-0.0000073(5)
U_{112}	0.0000169(7)	0.0000182(7)	0.0000172(7)
U_{113}	-0.0000022(4)	-0.0000023(4)	-0.0000023(4)
U_{122}	-0.0000270(11)	-0.0000275(11)	-0.0000271(11)
U_{223}	-0.0000148(9)	-0.0000151(9)	-0.0000148(9)
U_{133}	-0.0000022(3)	-0.0000024(3)	-0.0000023(3)
U_{233}	0.0000052(5)	0.0000060(5)	0.0000054(5)
U_{123}	0.0000005(4)	0.0000005(4)	0.0000005(4)

Table S14 4th order of the Gram-Charlier coefficients obtained for the SP8 data. Values higher than three standard uncertainties are highlighted in orange.

	rks-anh_nr	rhf-anh_rel	rks-anh_rel
U_{1111}	0.0000041(4)	0.0000045(4)	0.0000036(4)
U_{2222}	0.000010(2)	0.000014(2)	0.0000096(19)
U_{3333}	0.00000185(18)	0.00000196(18)	0.00000162(18)
U_{1112}	-0.00000166(19)	-0.00000160(19)	-0.00000152(19)
U_{1113}	0.00000070(14)	0.00000066(14)	0.00000065(14)
U_{1222}	-0.0000054(5)	-0.0000053(5)	-0.0000051(5)
U_{2223}	-0.0000029(4)	-0.0000028(4)	-0.0000027(4)
U_{1333}	0.00000080(9)	0.00000078(9)	0.00000077(9)
U_{2333}	-0.00000087(11)	-0.00000081(11)	-0.00000078(11)
U_{1122}	0.0000024(3)	0.0000029(3)	0.0000022(3)
U_{1133}	0.00000069(9)	0.00000077(9)	0.00000059(9)
U_{2233}	0.00000114(19)	0.00000143(19)	0.00000100(19)
U_{1123}	-0.00000042(9)	-0.00000035(9)	-0.00000035(9)
U_{1223}	0.00000209(15)	0.00000208(15)	0.00000205(15)
U_{1233}	-0.00000039(7)	-0.00000033(7)	-0.00000033(7)

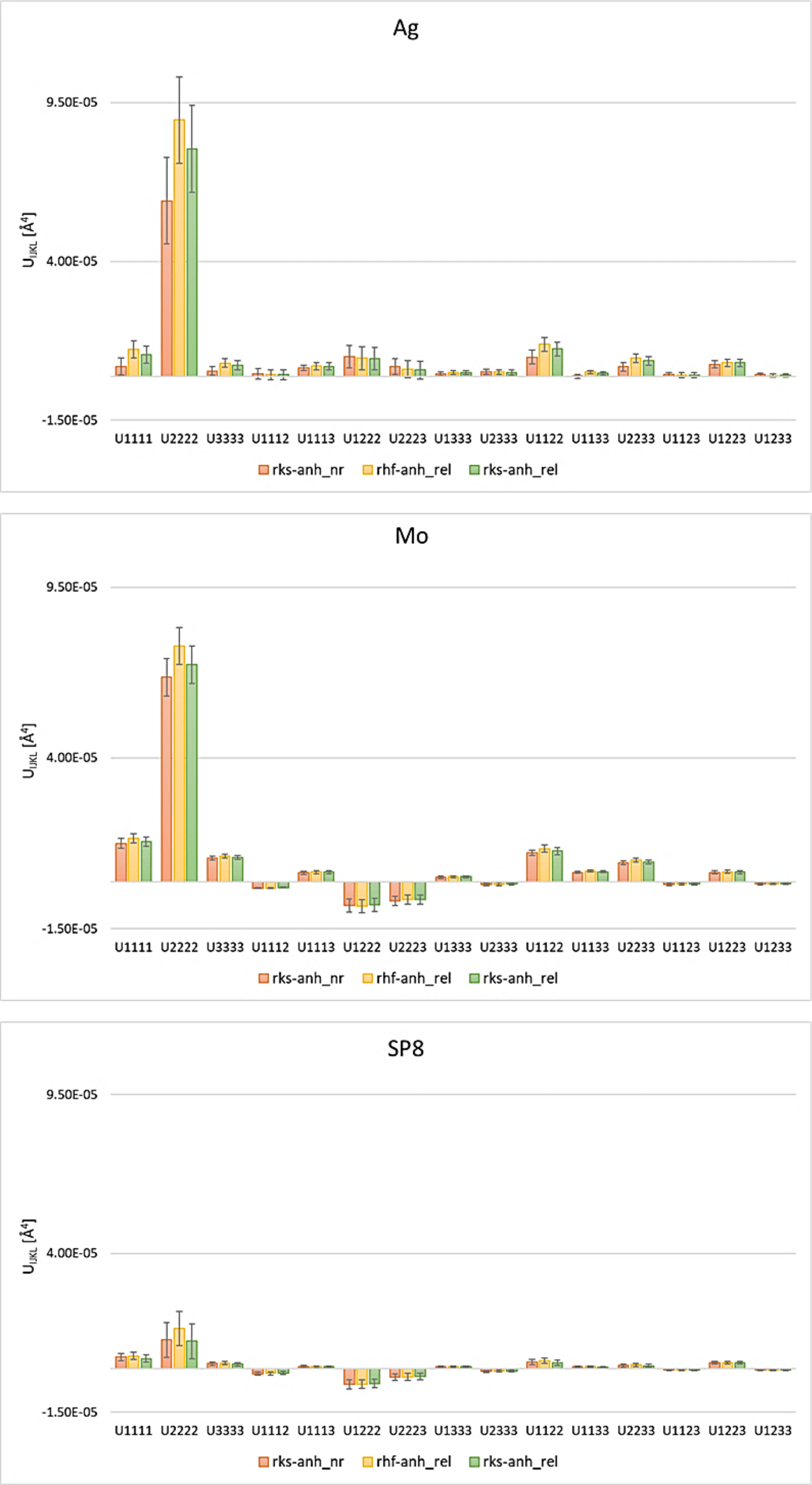

Figure S2 Plot of the numeric values of the 4th order G-C coefficients within three e.s.d.'s for Ag, Mo and SP8 data at different level of theory.

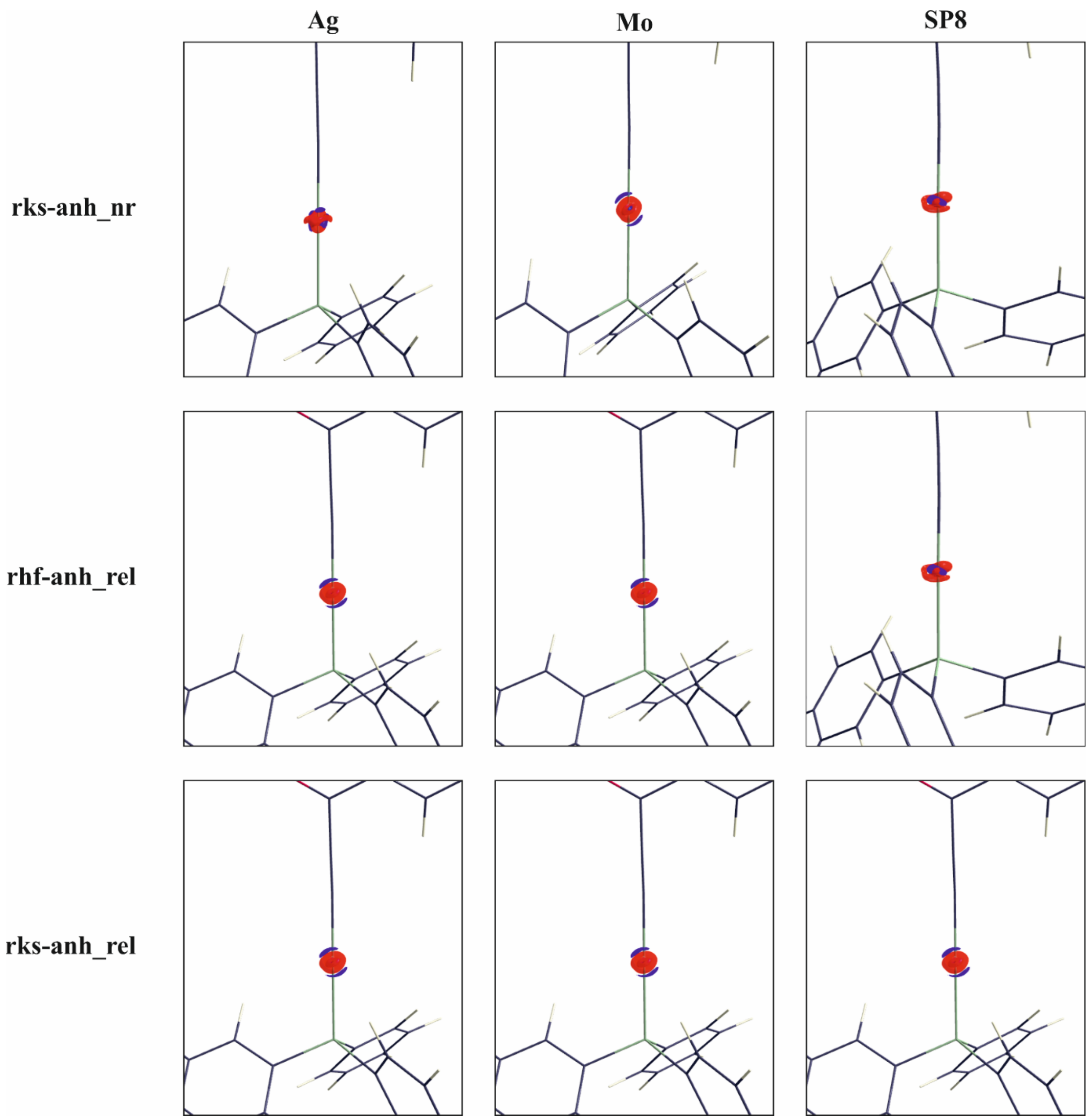

Figure S3 Graphical representation of the probability function of 3rd and 4th order of the Gram–Charlier coefficients of gold at the 50 % probability level for all considered anharmonic refinements.

Table S15 The difference between ADPs obtained from rks_nr, rhf_rel and rks_rel, representing effect of electron correlation (rks_rel – rhf_rel) and relativity (rks_rel – rks_nr)for Ag and Mo data.

Ag data					
Au	rks_nr	rhf_rel	rks_rel	ECORR	REL
U_{11}	0.01630(2)	0.01646(2)	0.01662(2)	0.00016	0.00032
U_{22}	0.02220(2)	0.02238(2)	0.02251(2)	0.00013	0.00031
U_{33}	0.01289(2)	0.01303(2)	0.01318(2)	0.00015	0.00030
U_{12}	0.00383(1)	0.00383(1)	0.00382(1)	-0.00001	-0.00001
U_{13}	0.00197(1)	0.00198(1)	0.00120(1)	0.00002	0.00002
U_{23}	0.00323(1)	0.00323(1)	0.00322(1)	-0.00001	-0.00001
			**Mo data**		
**Au**	**rks_nr**	**rhf_rel**	**rks_rel**	**ECORR**	**REL**
U11	0.01786(1)	0.01799(1)	0.01810(1)	0.00011	0.00024
U22	0.02396(2)	0.02411(2)	0.02422(2)	0.00011	0.00025
U33	0.01349(1)	0.01362(1)	0.01372(1)	0.00010	0.00023
U12	-0.004305(8)	-0.004307(8)	-0.004301(8)	0.00001	0.00000
U13	0.001988(8)	0.001992(8)	0.002005(8)	0.00001	0.00002
U23	-0.003720(7)	-0.003721(7)	-0.003715(8)	0.00001	0.00001

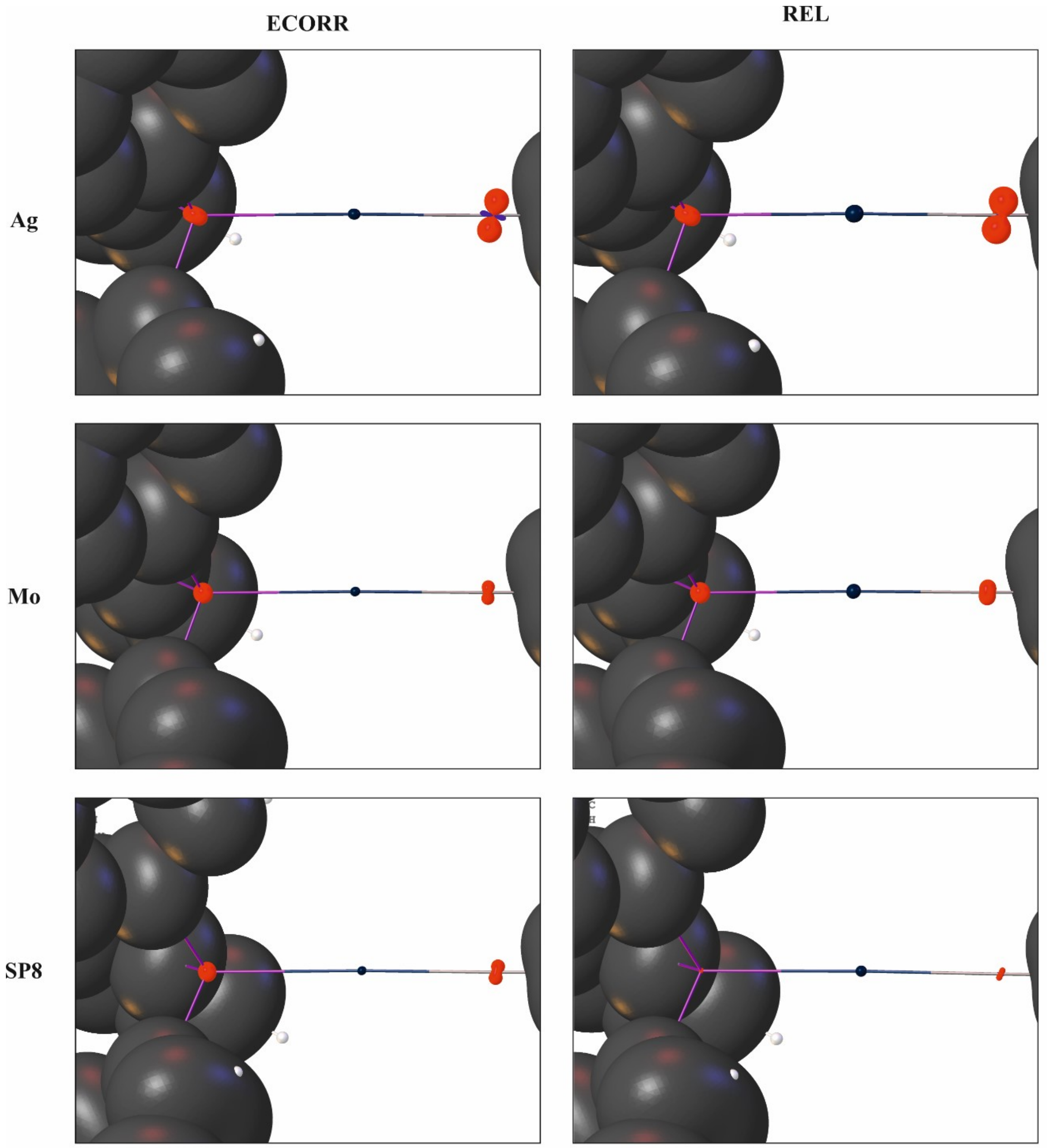

Figure S4 Graphical representation of the differences between ADPs obtained from rks_nr, rhf_rel and rks_rel, for Ag, Mo and SP8 data in the P–Au–C direction. The central atom is Au, left P and right C.

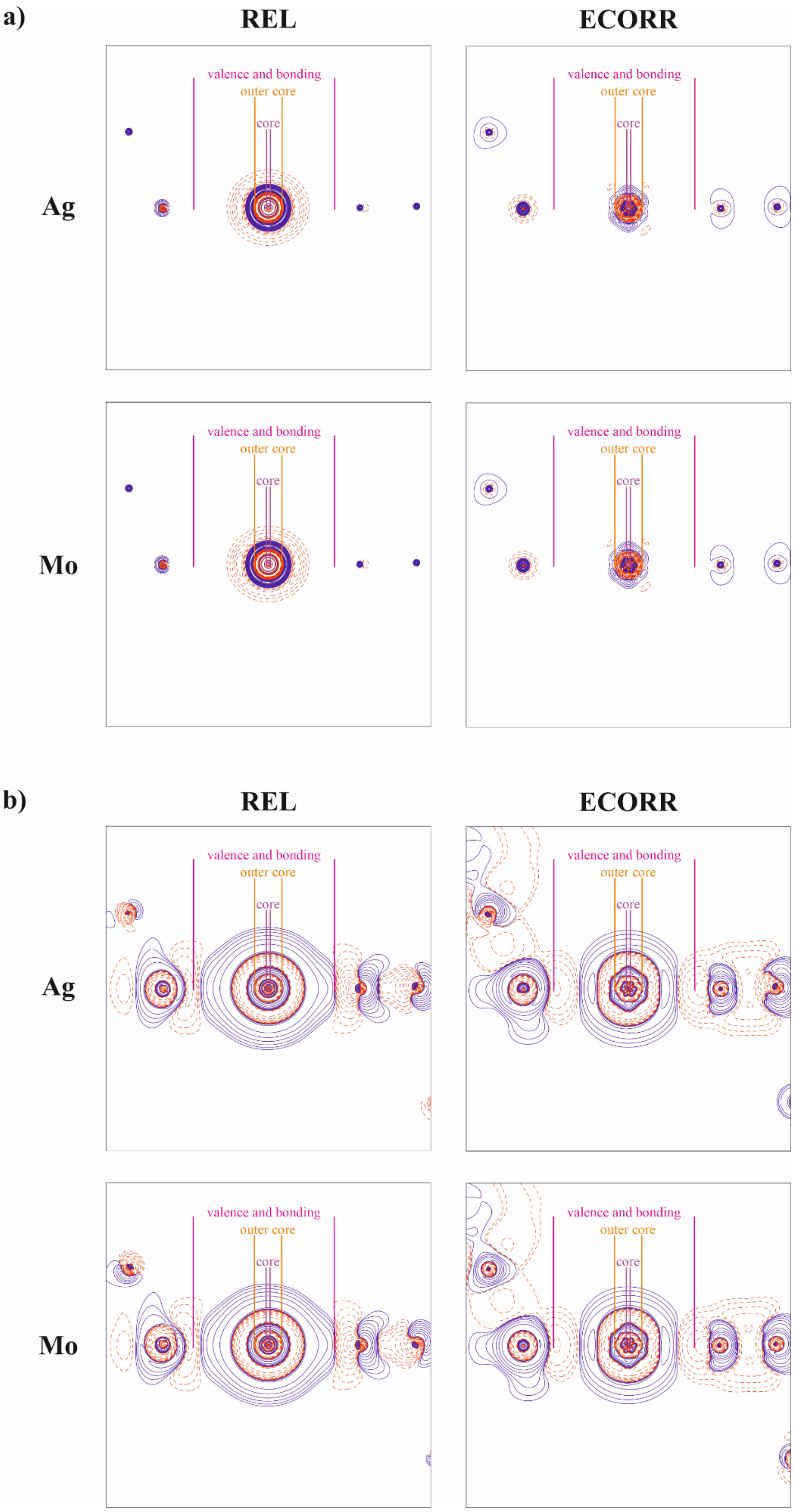

Figure S5 Difference maps of (a) the static electron density (contour ±0.01 e A−3) and (b) negative Laplacian (contour values in geometric order, starting value ±0.1, increments 2) in the plane of P–Au–C atoms exposing the effects of relativity (REL) and electron correlation (ECORR) for Ag and Mo data. Values of the positive and negative difference densities are denoted by the blue, solid and red, dashed lines, respectively.

Table S16 **Selected BCP topological properties of** Au–C bond resulting from HAR performed against Ag and Mo data. “dev” represents changes in ρ(r) and ∇2ρ(r) arising from REL, ECORR and ANH and are expressed in percentages.

					**Ag data**						
	**ρ(r) eÅ^{-3}**	**dev %**	**$\nabla^2\rho(r)$ eÅ^{-5}**	**dev %**	**V_r [Haa$_0^{-3}$]**	**G_r [Haa$_0^{-3}$]**	**H_r [Haa$_0^{-3}$]**	**$\lvert V_r\rvert/G_r$**	**ε**	**r_{Au-BCP} [Å]**	**r_{Au-P} [Å]**
**rks-anh_nr**	0.930	- 2.1	8.389	12.0	-0.1943	0.1407	-0.0536	1.38	0.02	1.09	1.9878(8)
**rhf-anh_rel**	0.972	2.3	8.291	10.7	-0.2133	0.1497	-0.0637	1.43	0.00	1.06	1.9886(10)
**rks-rel**	0.971	2.2	6.902	-7.9	-0.1950	0.1333	-0.0617	1.46	0.02	1.07	1.9892(10)
**rks-anh_rel**	0.950		7.491		-0.1964	0.1371	-0.0593	1.43	0.01	1.06	1.9884(10)
					Mo data						
	ρ(r) eÅ^{-3}	dev %	**∇^2**ρ(r) eÅ^{-5}	dev %	V_r [Haa$_0^{-3}$]	G_r [Haa$_0^{-3}$]	H_r [Haa$_0^{-3}$]	$\lvert V_r\rvert/G_r$	ε	r_{Au-BCP} [Å]	r_{Au-P} [Å]
**rks-anh_nr**	0.932	- 2.5	9.007	19.5	-0.1977	0.1456	-0.0521	1.36	0.00	1.07	1.9854(7)
**rhf-anh_rel**	0.978	2.3	8.341	10.7	-0.2151	0.1508	-0.0643	1.43	0.00	1.05	1.9856(7)
**rks-rel**	0.976	2.1	6.974	-7.5	-0.1969	0.1346	-0.0623	1.46	0.02	1.07	1.9851(9)
**rks-anh_rel**	0.956		7.537		-0.1983	0.1382	-0.0600	1.43	0.01	1.06	1.9854(7)

Table S17 Selected BCP topological properties of Au–P bond resulting from HAR performed against Ag and Mo data. "dev" represents changes in ρ(r) and $\nabla^2\rho(r)$ arising from REL, ECORR and ANH and are expressed in percentages.

						**Ag data**					
	**ρ(r) eÅ^{-3}**	**dev %**	**∇^2ρ(r) eÅ^{-5}**	**dev %**	**V_r [Haa$_0^{-3}$]**	**G_r [Haa$_0^{-3}$]**	**H_r [Haa$_0^{-3}$]**	**$\lvert V_r\rvert/G_r$**	**ε**	**$r_{Au\text{-}BCP}$ [Å]**	**$r_{Au\text{-}P}$ [Å]**
**rks-anh_nr**	0.739	-5.1	0.728	-9.7	-0.1170	0.0623	-0.0547	1.88	0.00	1.20	2.2742(2)
**rhf-anh_rel**	0.786	0.9	1.399	73.6	-0.1319	0.0732	-0.0587	1.80	0.01	1.16	2.2741(2)
**rks-rel**	0.803	3.1	2.237	177.5	-0.1381	0.0806	-0.0574	1.71	0.04	1.15	2.2733(3)
**rks-anh_rel**	0.779		0.806		-0.1244	0.0664	-0.0580	1.87	0.01	1.17	2.2742(2)
						**Mo data**					
	**ρ(r) eÅ^{-3}**	**dev %**	**∇^2ρ(r) eÅ^{-5}**	**dev %**	**V_r [Haa$_0^{-3}$]**	**G_r [Haa$_0^{-3}$]**	**H_r [Haa$_0^{-3}$]**	**$\lvert V_r\rvert/G_r$**	**ε**	**$r_{Au\text{-}BCP}$ [Å]**	**$r_{Au\text{-}P}$ [Å]**
**rks-anh_nr**	0.740	-5.1	0.744	-11.8	-0.1175	0.0626	-0.0549	1.88	0.00	1.20	2.2726(2)
**rhf-anh_rel**	0.788	1.0	1.431	69.5	-0.1326	0.0737	-0.0589	1.80	0.01	1.16	2.2726(2)
**rks-rel**	0.805	3.2	2.230	164.2	-0.1384	0.0808	-0.0576	1.71	0.05	1.15	2.2725(2)
**rks-anh_rel**	0.780		0.844		-0.1250	0.0669	-0.0581	1.87	0.01	1.17	2.2726(2)

Table S18 AIM charges resulting from HARs performed against Ag data.

atom	rks-anh_nr	rhf-anh_rel	rks_rel	rks-anh_rel
Au1	1.041	0.452	0.184	0.618
P1	2.231	2.448	1.992	2.120
Cl1	-0.252	-0.326	0.146	-0.250
O1	-1.140	-1.321	-0.962	-1.138
C1	-0.161	-0.092	-0.034	-0.170
C2	1.029	-0.509	-0.124	-0.332
C3	-0.042	1.224	1.026	1.030
C4	0.004	-0.053	0.370	-0.044
C5	0.054	0.001	0.064	0.003
C6	0.080	0.077	0.165	0.054
C7	0.045	0.086	0.402	0.079
C8	0.078	0.071	0.173	0.043
C9	-0.592	0.107	0.093	0.079
C10	0.092	-0.690	-0.662	-0.594
C11	0.028	0.122	0.023	0.096
C12	0.118	0.034	0.054	0.024
C13	0.095	0.162	0.078	0.120
C14	0.048	0.158	0.037	0.108
C15	-0.595	0.036	0.018	0.033
C16	0.078	-0.692	-0.654	-0.589
C17	0.006	0.080	0.057	0.072
C18	0.021	0.010	0.021	0.008
C19	-0.369	0.041	0.043	0.021
C20	-0.008	-0.012	0.014	-0.010
C21	0.040	0.047	-0.007	0.037
C22	-0.491	-0.429	-0.646	-0.350
C23	0.128	0.220	0.007	0.228

C24	0.002	0.005	0.017	-0.009
C25	0.205	0.383	0.015	0.346
C26	0.093	0.232	0.006	0.218
C27	0.037	0.006	-0.001	0.004
H5	0.028	0.043	0.031	0.030
H6	0.051	0.069	0.028	0.055
H8	0.011	0.012	0.012	0.012
H9	-0.019	-0.027	-0.007	-0.019
H11	-0.009	-0.004	-0.004	0.001
H12	-0.040	-0.055	-0.002	-0.040
H13	0.023	0.020	-0.011	0.024
H14	-0.039	-0.061	-0.045	-0.036
H15	-0.021	-0.026	-0.009	-0.021
H17	0.007	0.022	0.002	0.018
H18	0.007	0.010	0.007	0.008
H19	-0.002	-0.008	0.010	-0.003
H20	0.020	0.024	0.000	0.022
H21	0.010	0.016	0.002	0.012
H23	-0.024	-0.030	0.018	-0.020
H24	0.011	0.011	0.017	0.011
H25	0.051	0.067	0.023	0.054
H26	0.019	0.018	-0.003	0.023
H27	0.010	0.017	0.017	0.012

Table S19 AIM charges resulting from HARs performed against Mo data.

atom	rks-anh_nr	rhf-anh_rel	rks_rel	rks-anh_rel
Au1	1.021	0.445	0.191	0.591
P1	2.234	2.448	1.995	2.122
Cl1	-0.251	-0.324	0.151	-0.249
O1	-1.141	-1.324	-0.988	-1.139
C1	-0.163	-0.090	-0.027	-0.168
C2	-0.370	-0.510	-0.122	-0.332
C3	1.024	1.222	1.027	1.026
C4	-0.041	-0.052	0.365	-0.042
C5	0.008	0.009	0.055	0.006
C6	0.039	0.055	0.182	0.039
C7	0.086	0.093	0.401	0.085
C8	0.044	0.068	0.167	0.044
C9	0.033	0.039	0.106	0.032
C10	-0.595	-0.700	-0.666	-0.600
C11	0.067	0.077	0.005	0.067
C12	0.055	0.077	0.065	0.055
C13	0.083	0.101	0.026	0.083
C14	0.073	0.118	0.039	0.083
C15	0.051	0.039	0.028	0.038
C16	-0.590	-0.686	-0.660	-0.587
C17	0.078	0.077	0.065	0.072
C18	0.023	0.044	0.051	0.025
C19	0.021	0.032	0.026	0.021
C20	0.012	0.011	0.026	0.007
C21	0.013	0.009	-0.009	0.010
C22	-0.477	-0.413	-0.651	-0.337
C23	0.142	0.245	0.045	0.250

C24	0.002	0.003	0.030	-0.007
C25	0.234	0.418	0.047	0.370
C26	0.103	0.248	0.028	0.241
C27	0.046	0.018	0.027	0.012
H5	0.030	0.043	0.030	0.031
H6	0.063	0.085	0.020	0.067
H8	0.015	0.020	0.014	0.016
H9	0.015	0.027	-0.010	0.016
H11	0.005	0.017	0.006	0.015
H12	-0.053	-0.079	-0.004	-0.056
H13	0.051	0.066	0.032	0.052
H14	-0.018	-0.029	-0.040	-0.016
H15	-0.024	-0.026	-0.018	-0.025
H17	0.006	0.020	-0.010	0.016
H18	-0.007	-0.011	-0.016	-0.007
H19	-0.005	-0.010	0.020	-0.007
H20	0.010	0.012	0.000	0.012
H21	0.024	0.035	0.000	0.026
H23	-0.030	-0.041	-0.010	-0.027
H24	0.007	0.006	0.000	0.008
H25	0.030	0.040	-0.004	0.034
H26	0.015	0.015	-0.025	0.018
H27	0.004	0.009	-0.007	0.005

Table S20 AIM charges resulting from HARs performed against SP8 data.

atom	rks-anh_nr	rhf-anh_rel	rks_rel	rks-anh_rel
Au1	0.994	0.430	0.189	0.557
P1	2.223	2.436	1.986	2.110
Cl1	-0.252	-0.325	0.142	-0.250
O1	-1.137	-1.320	-0.975	-1.134
C1	-0.168	-0.094	-0.037	-0.170
C2	-0.368	-0.505	-0.127	-0.328
C3	1.019	1.217	1.023	1.022
C4	-0.041	-0.053	0.372	-0.043
C5	0.018	0.026	0.078	0.017
C6	0.053	0.071	0.185	0.052
C7	0.078	0.083	0.403	0.078
C8	0.058	0.081	0.185	0.056
C9	0.024	0.025	0.082	0.023
C10	-0.587	-0.697	-0.657	-0.596
C11	0.080	0.096	0.023	0.079
C12	0.034	0.041	0.035	0.033
C13	0.115	0.135	0.060	0.112
C14	0.054	0.086	0.019	0.061
C15	0.049	0.037	0.016	0.036
C16	-0.589	-0.691	-0.661	-0.587
C17	0.063	0.061	0.048	0.057
C18	0.030	0.047	0.069	0.031
C19	0.021	0.036	0.031	0.020
C20	0.006	-0.001	0.031	0.000
C21	0.030	0.029	0.012	0.026
C22	-0.470	-0.413	-0.650	-0.328
C23	0.155	0.274	0.045	0.273

C24	0.034	0.039	0.054	0.022
C25	0.216	0.378	0.010	0.352
C26	0.107	0.274	0.019	0.254
C27	0.042	-0.003	0.014	0.004
H5	0.024	0.032	0.023	0.024
H6	0.053	0.076	0.013	0.057
H8	0.007	0.013	0.006	0.007
H9	0.021	0.037	0.002	0.021
H11	-0.001	0.006	-0.001	0.009
H12	-0.047	-0.064	0.011	-0.048
H13	0.021	0.030	0.004	0.022
H14	-0.009	-0.012	-0.030	-0.006
H15	-0.017	-0.016	-0.005	-0.018
H17	0.016	0.033	0.001	0.027
H18	-0.003	-0.007	-0.024	-0.003
H19	-0.007	-0.011	0.012	-0.008
H20	0.011	0.014	-0.006	0.014
H21	0.015	0.024	-0.012	0.017
H23	-0.033	-0.039	-0.009	-0.029
H24	-0.007	-0.014	-0.011	-0.008
H25	0.046	0.065	0.019	0.052
H26	0.019	0.019	-0.021	0.021
H27	0.004	0.012	0.002	0.006

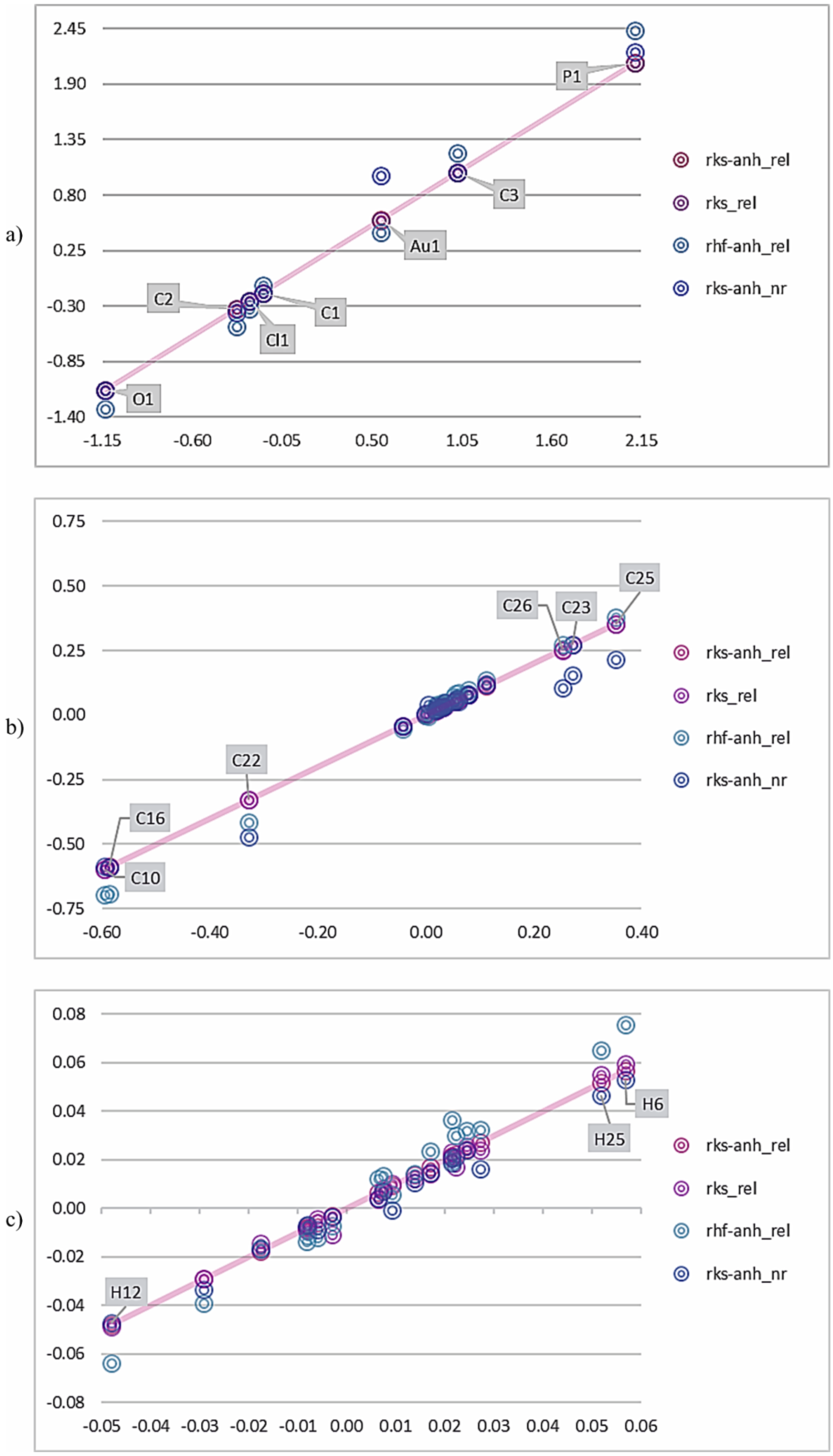

Figure S6 Plot of atomic charges obtained from QTAIM analysis after HARs against SP8 data showing changes arising from ANH, REL and ECORR. Pink line represents y=x plot.

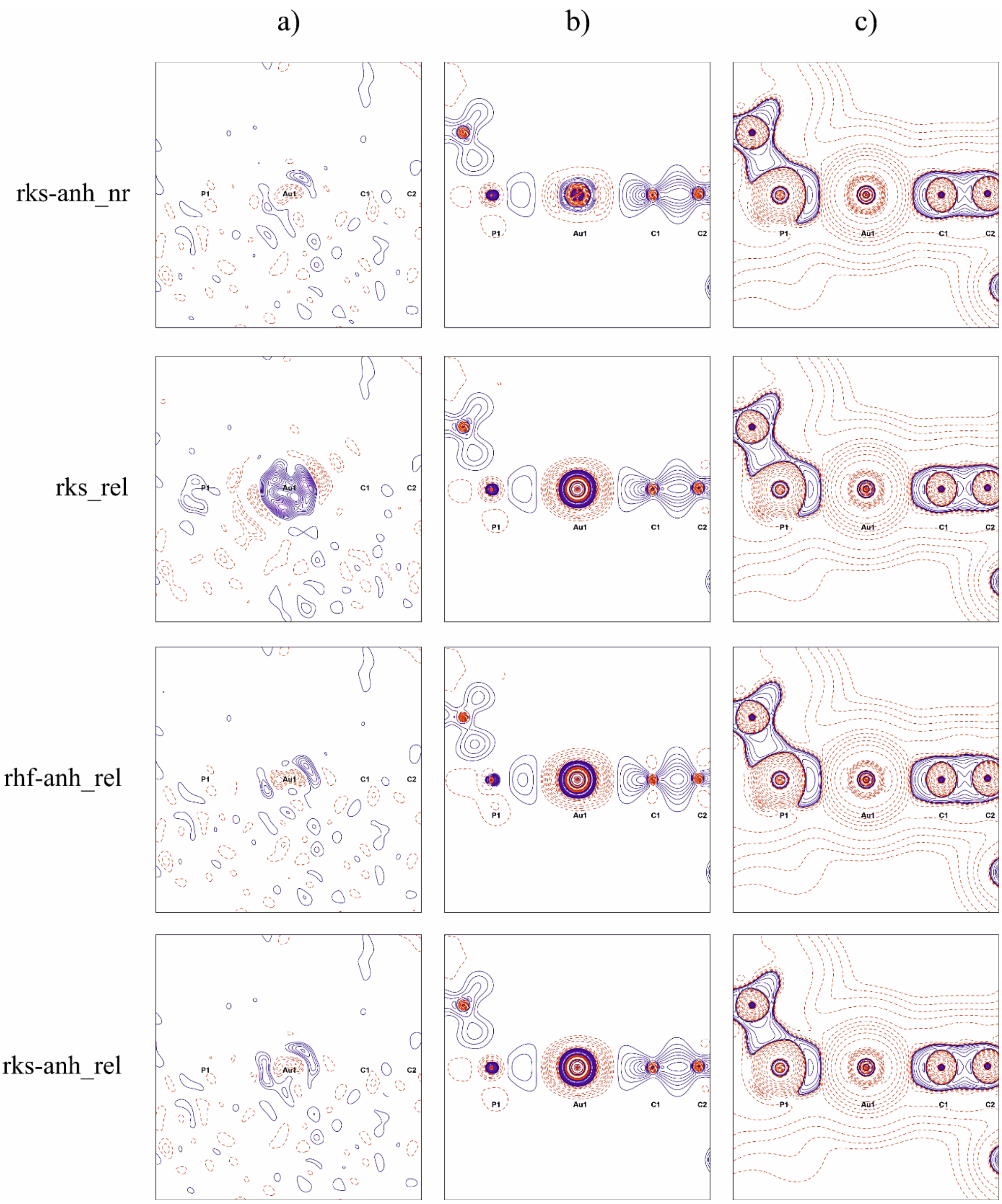

Figure S7 (a) Residual density, (b) deformation density and (c) negative Laplacian maps resulting from HAR performed against Ag data. Contour level: 0.1eÅ^{-3} (a and b) and 0.1eÅ^{-3} (c). Colours: blue – positive, red – negative.

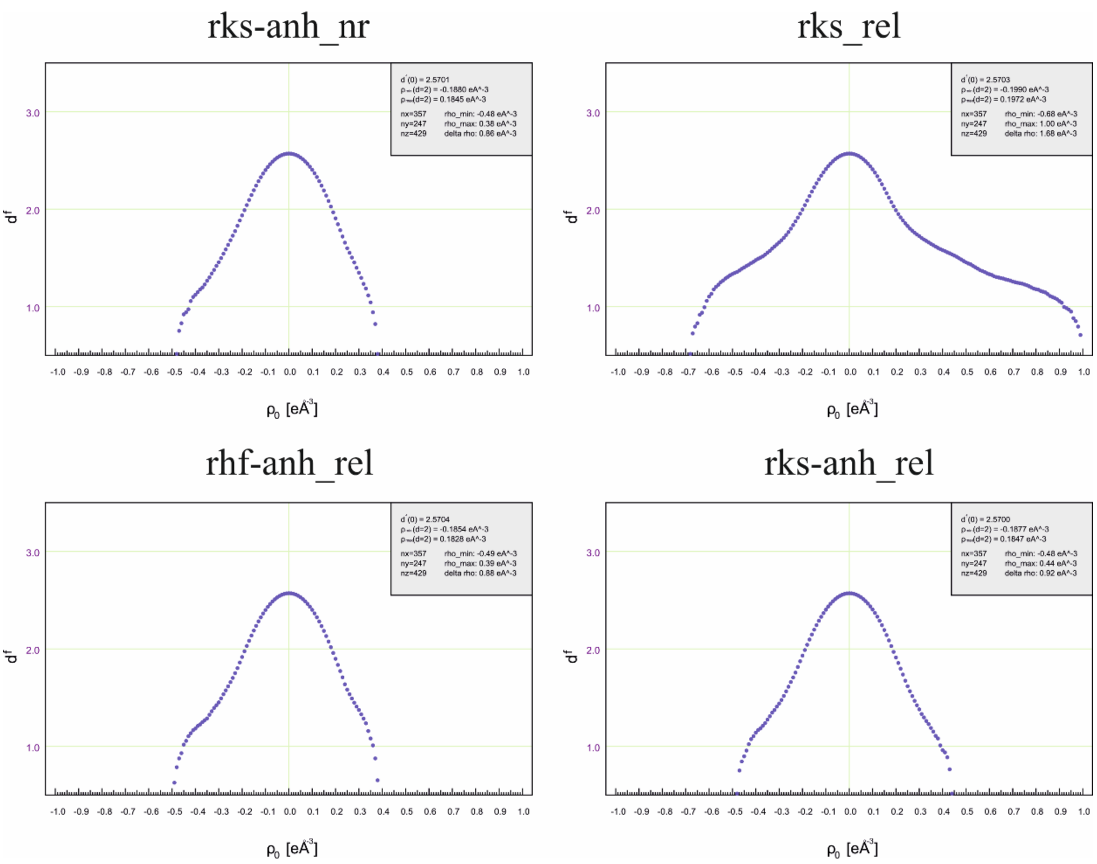

Figure S8 The fractal dimension plots of the residual electron density resulting from HAR performed against Ag data.

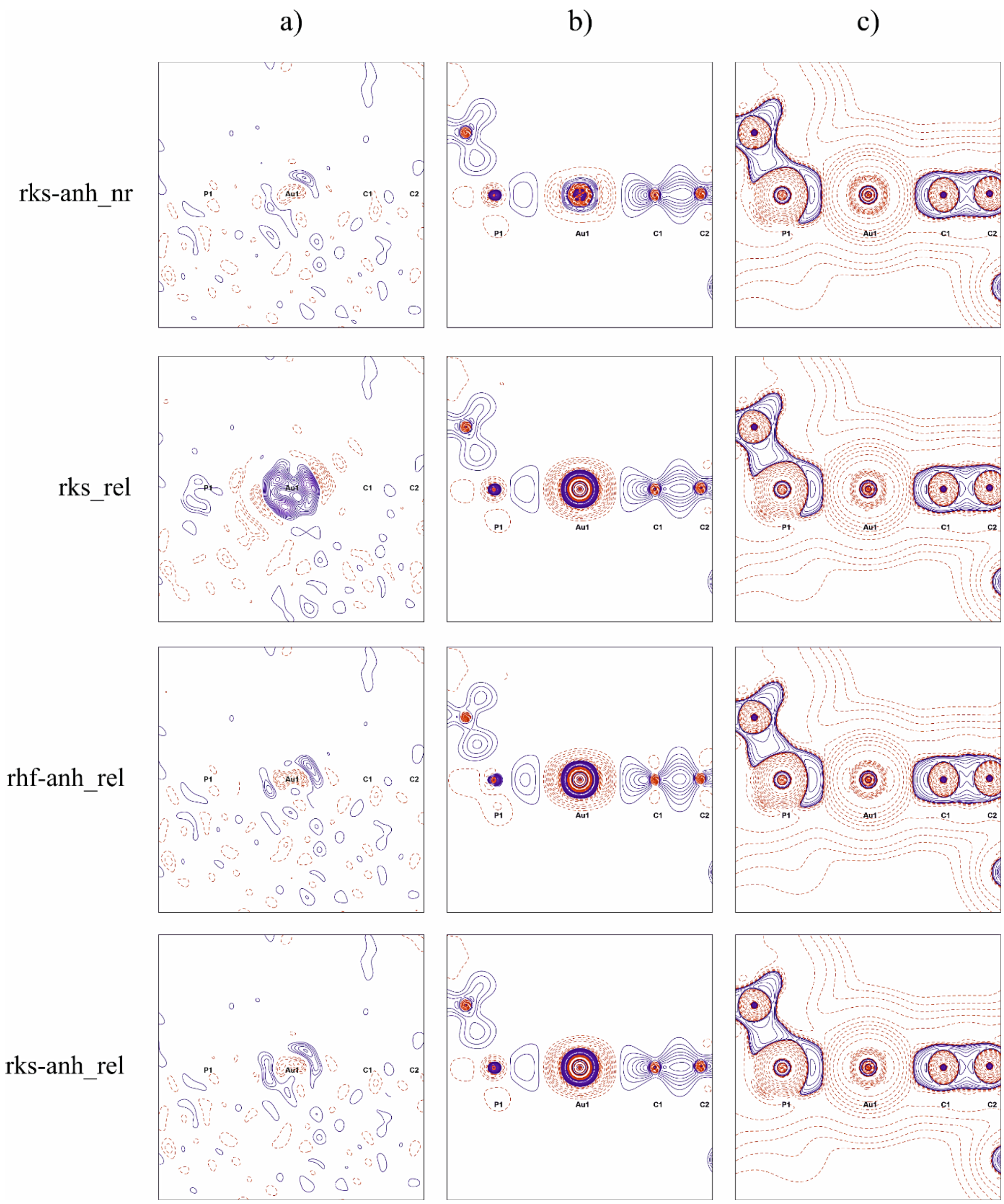

Figure S9 (a) Residual density, (b) deformation density and (c) negative Laplacian maps resulting from HAR performed against Mo data. Contour level: 0.1eÅ^{-3} (a and b) and 0.1eÅ^{-3} (c). Colours: blue – positive, red – negative.

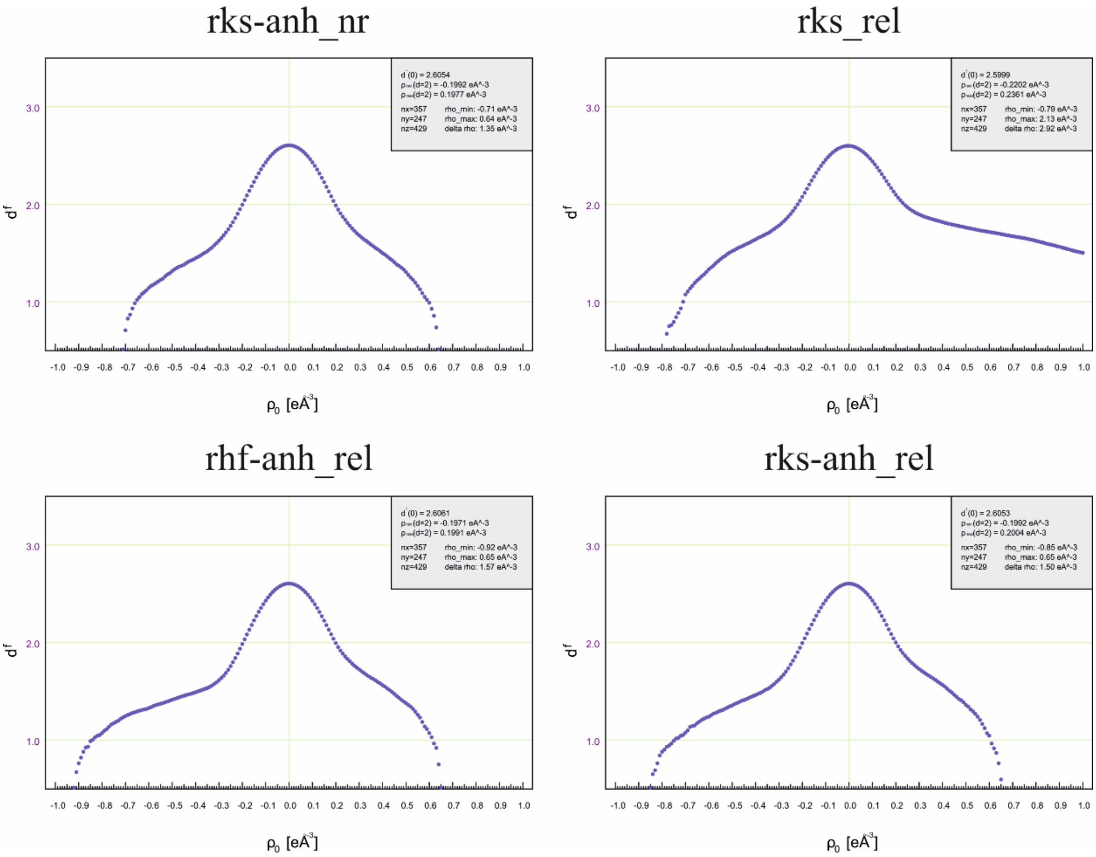

Figure S10 The fractal dimension plots of the residual electron density for resulting from HAR performed against Mo data.

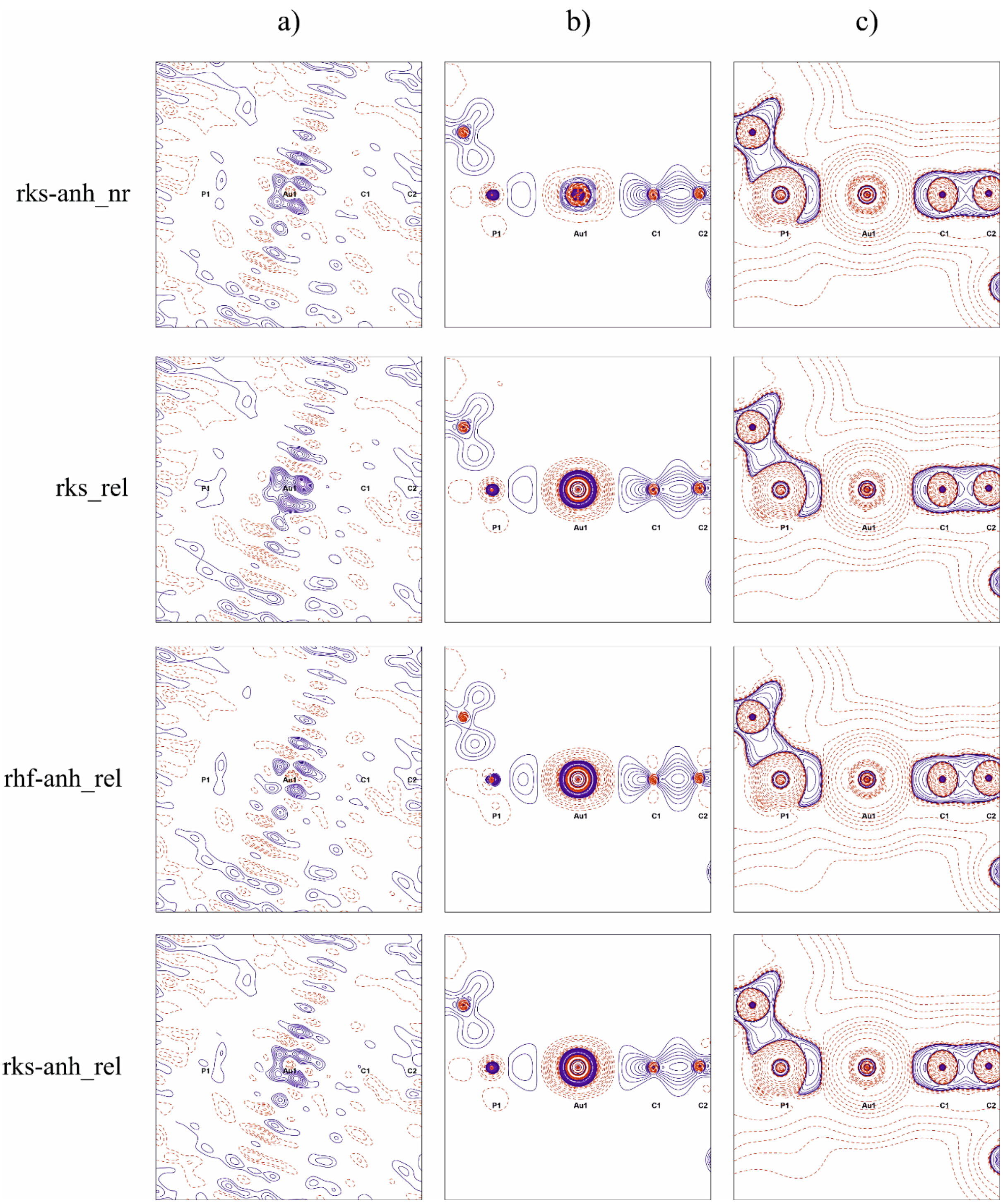

Figure S11(a) Residual density, (b) deformation density and (c) negative Laplacian maps resulting from HAR performed against SP8 data. Contour level: 0.1eÅ^{-3} (a and b) and 0.1eÅ^{-3} (c). Colours: blue – positive, red – negative.

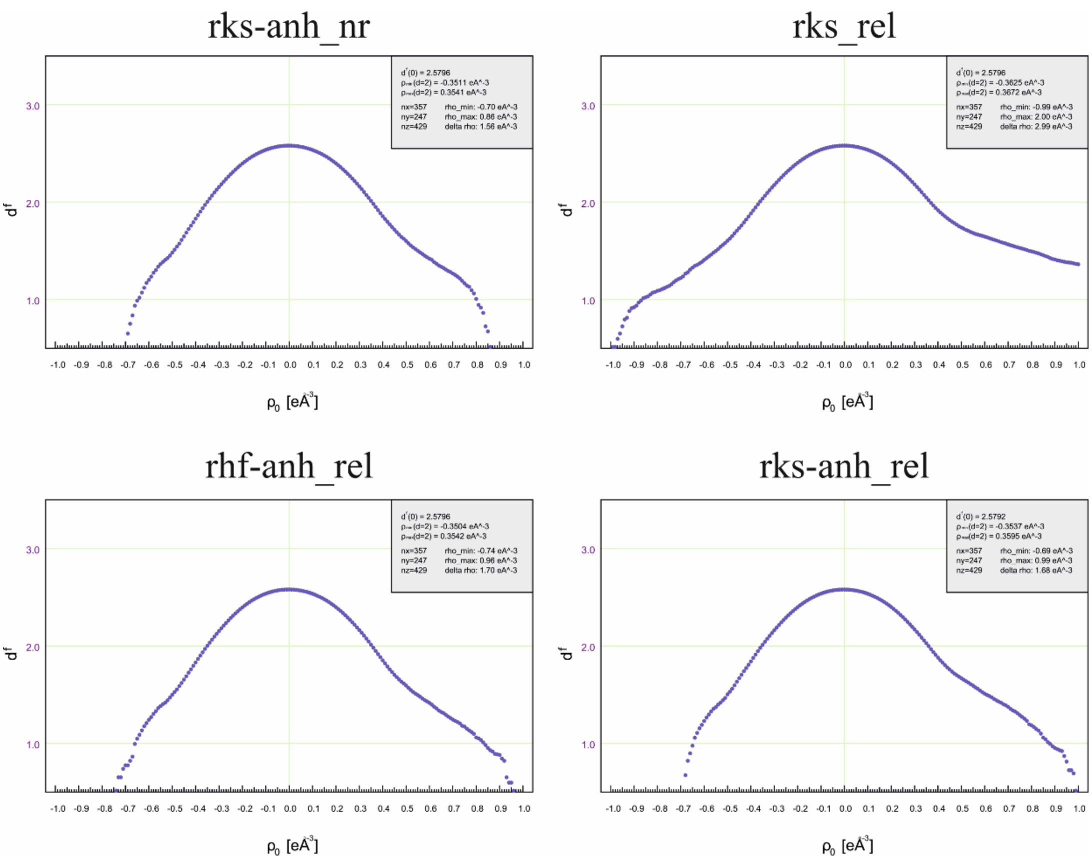

Figure S12 The fractal dimension plots of the residual electron density resulting from HAR performed against SP8 data.

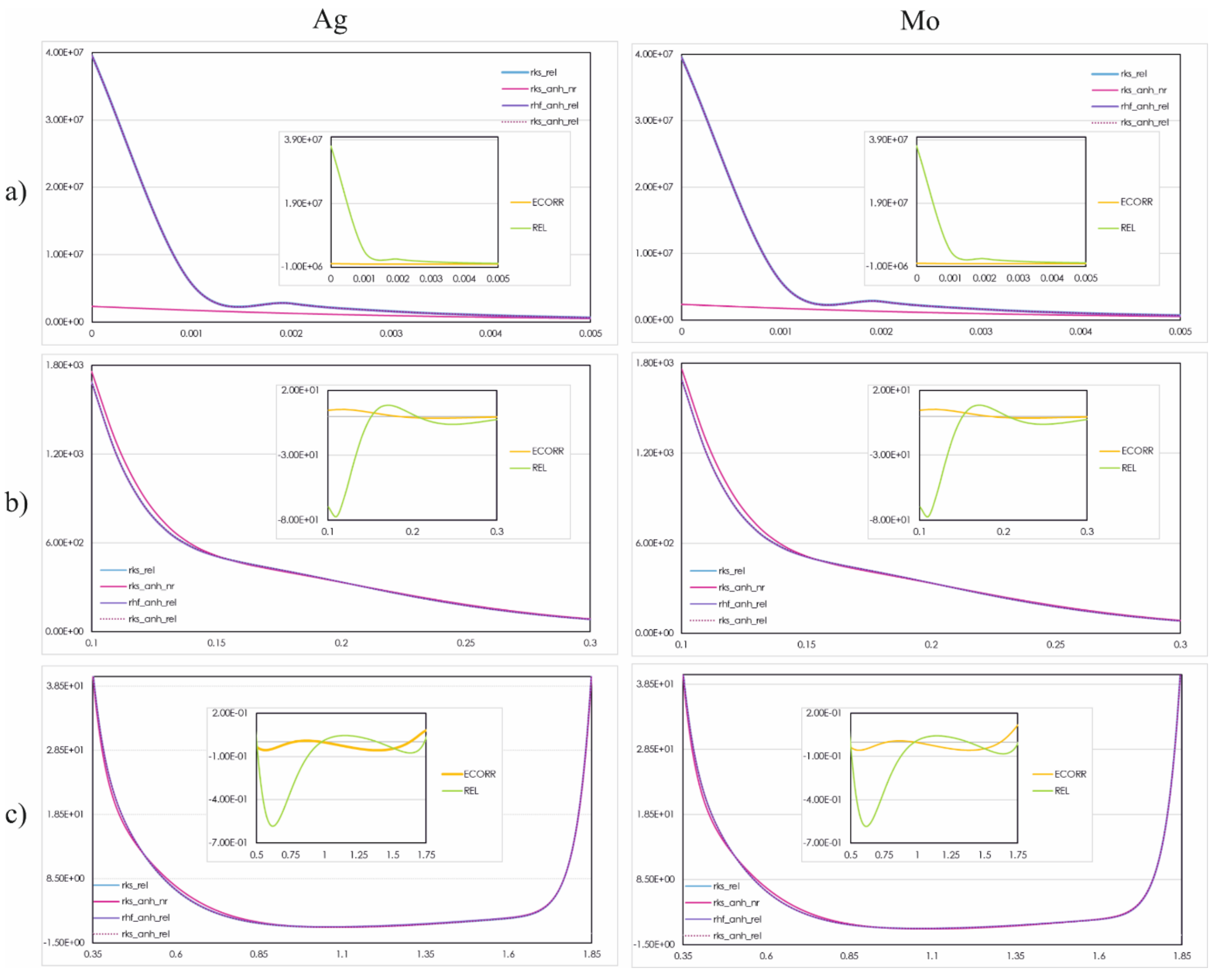

Figure S131D plots of electron density (y-axis, in eÅ-3) as a function of the Au−C bond distance (x-axis, in Å) resulting from HARs performed against Ag and Mo data. The subplots represents 1D difference plots resulting from the relativistic effects and electron correlation.

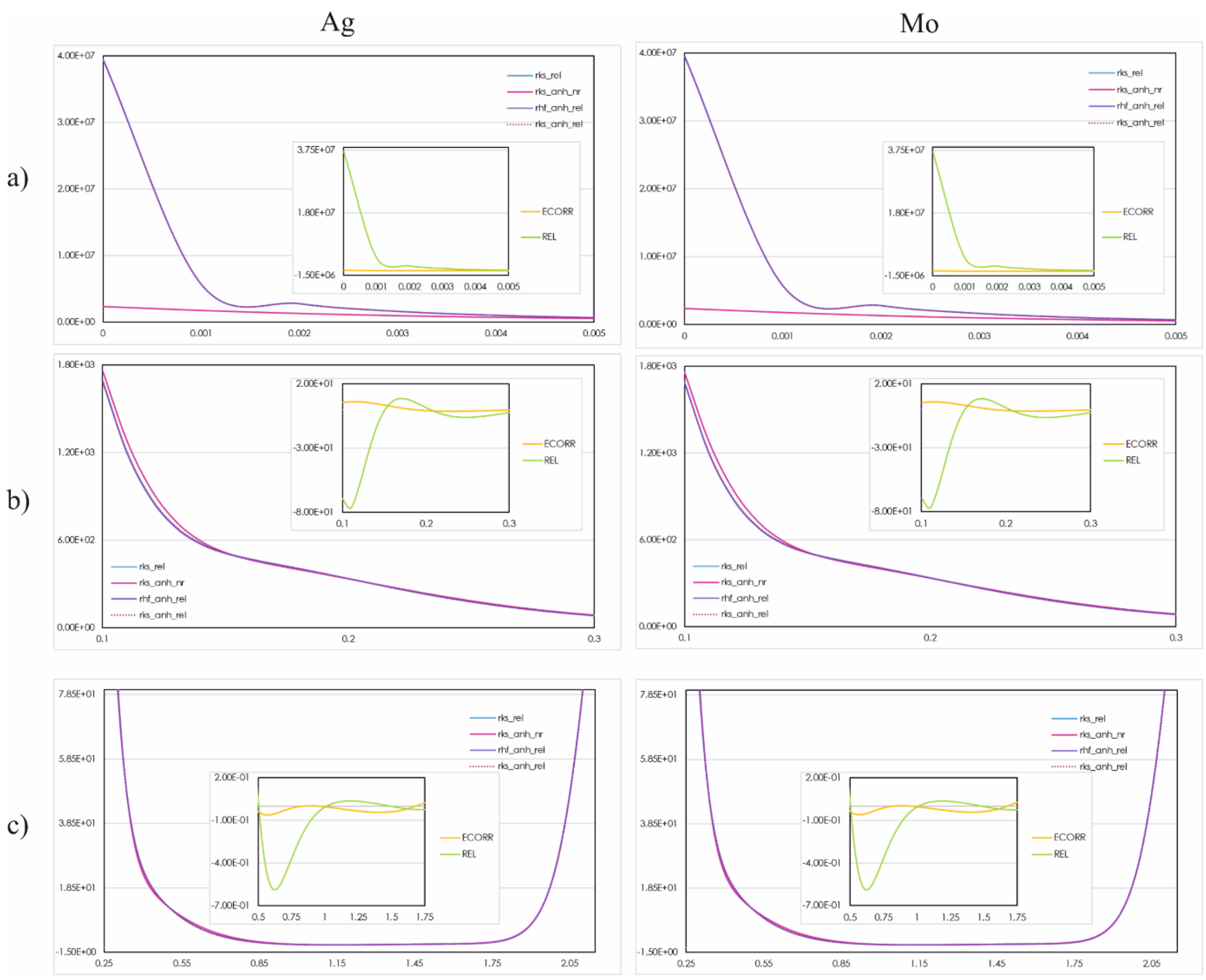

Figure S14 1D plots of electron density (y-axis, in eÅ-3) as a function of the Au−P bond distance (x-axis, in Å) resulting from HARs performed against Ag and Mo data. The subplots represents 1D difference plots resulting from the relativistic effects and electron correlation.

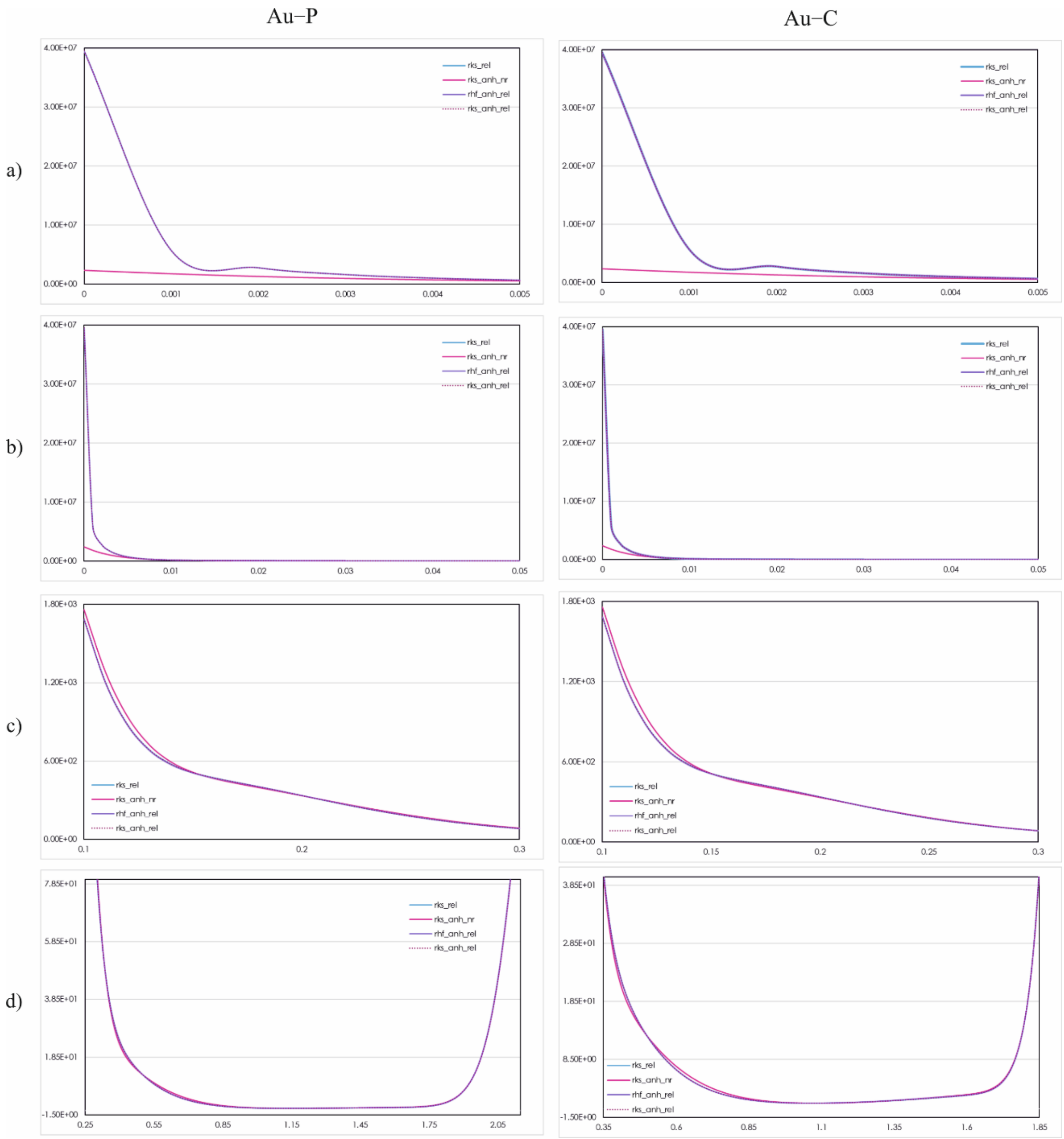

Figure S151D plots of electron density (y-axis, in eÅ-3) as a function of the Au–P and Au–C bond distance (x-axis, in Å) resulting from HARs performed against SP8 data.

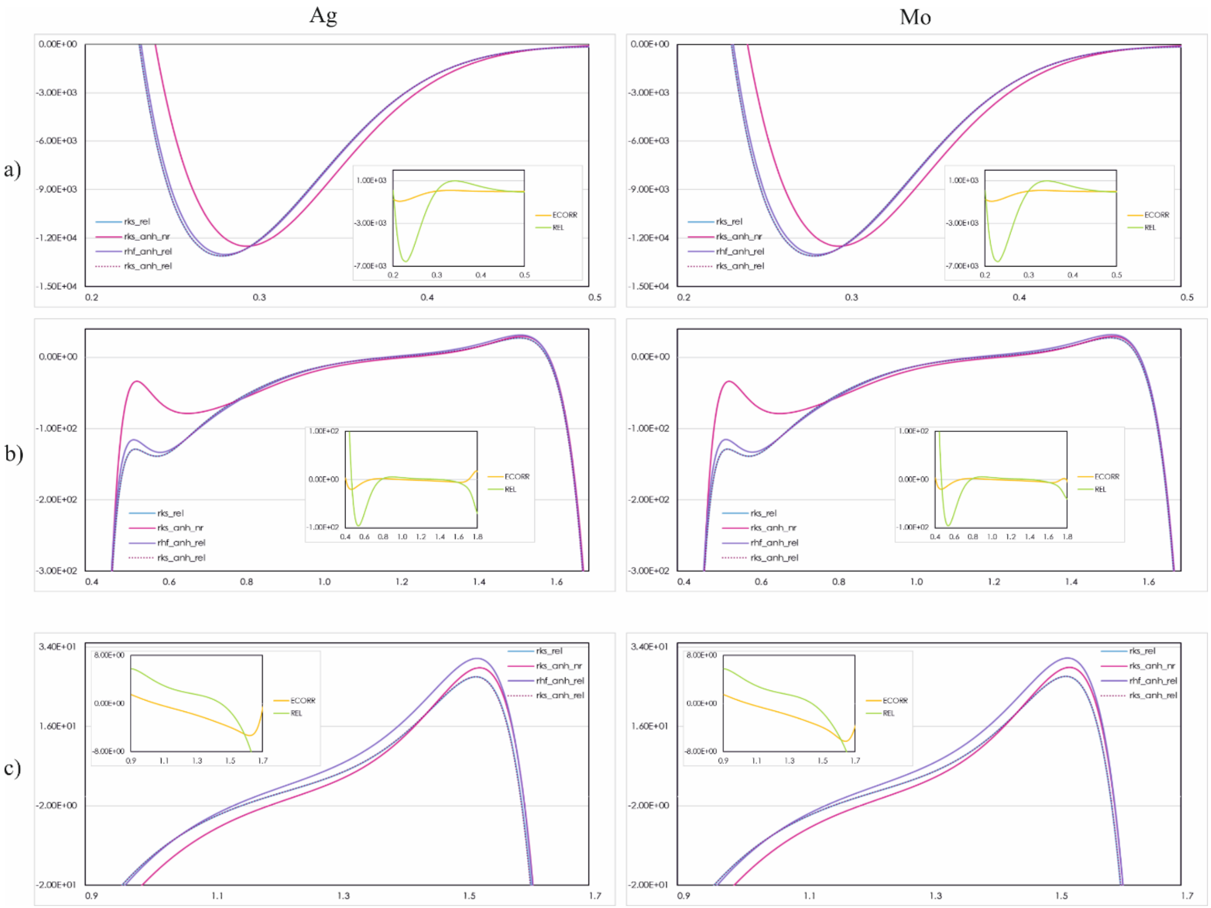

Figure S161D plots of negative Laplacian (y-axis, in eÅ^{-5}) as a function of the Au−C bond distance (x-axis, in Å) resulting from performed HARs against Ag and Mo data. The subplots show difference electron densities resulting from the relativistic and electron correlation effects.

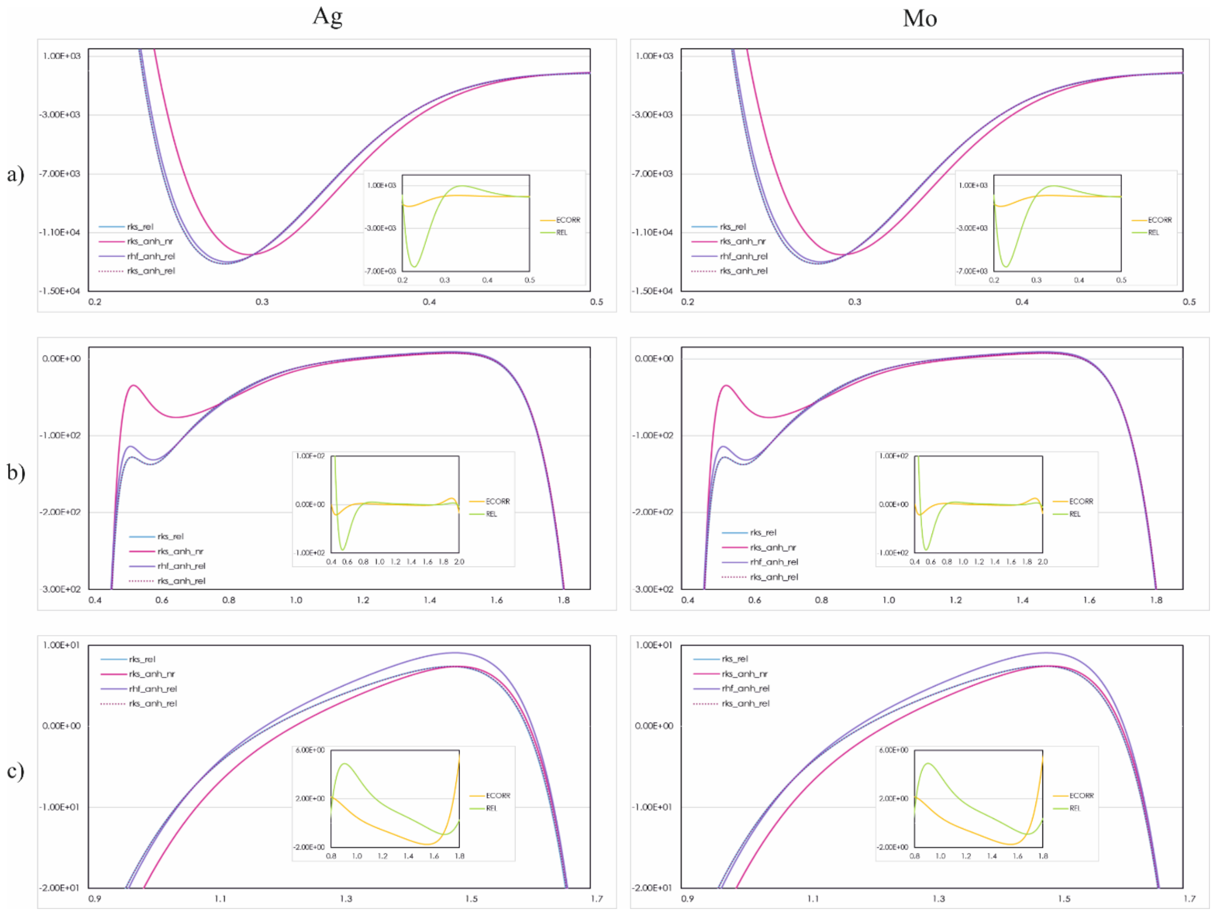

Figure S17 1D plots of negative Laplacian (y-axis, in $e\text{Å}^{-5}$) as a function of the Au−P bond distance (x-axis, in Å) resulting from performed HARs against Ag and Mo data. The subplots show difference electron densities resulting from the relativistic and electron correlation effects.
